# Assessing PrEP Initiation and Adherence Among High-Risk, Sexually Active Adolescents and Young Adults: A Population-Based Prospective Study Across Diverse Service Delivery Models in a High HIV Prevalent District in South Africa

**DOI:** 10.1007/s10461-025-04719-6

**Published:** 2025-05-02

**Authors:** Edward Nicol, Wisdom Basera, Eunice Turawa, Carl Lombard, Noluntu Funani, Dakalo Chavhalala, the SHeS’Cap-PrEP group

**Affiliations:** 1 Burden of Disease Research Unit, South African Medical Research Council, Cape Town, South Africa; 2 Division of Health Systems and Public Health, Stellenbosch University, Cape Town, South Africa; 3 School of Public Health and Family Medicine, University of Cape Town, Cape Town, South Africa; 4 Biostatistics Research Unit, South African Medical Research Council, Cape Town, South Africa; 5 TBHIV Care, Pietermaritzburg, KwaZulu Natal South Africa

**Keywords:** HIV, High-risk, PrEP uptake, PrEP adherence, Pre-exposure prophylaxis, AYAs, AGYW, ABYM, Sexually active, South Africa

## Abstract

Pre-exposure prophylaxis (PrEP) is an effective HIV prevention strategy for high-risk populations, including adolescent girls and young women (AGYW) and adolescent boys and young men (ABYM). While various PrEP-delivery models exist in South Africa, little is known about optimizing PrEP-uptake and adherence for AGYW and ABYM. This study explored preferred and feasible PrEP care models to enhance initiation and adherence among at-risk adolescents and young adults (AYAs) in a high-HIV prevalent rural district in South Africa. We conducted a longitudinal, population-based cohort study from August 2021 to July 2022 across 22 service delivery points (SDPs) in KwaZulu-Natal. High-risk, sexually active HIV-negative AGYW (15–24 years) and ABYM (15–35 years) were recruited from clinics, schools, and community-based youth zones. PrEP-uptake and adherence, based on national guidelines, were assessed using Pearson’s Chi square test and binomial regression. Of 2,772 HIV-negative AYA, 781 (28%) initiated PrEP. Despite 65% being classified as high risk for HIV (*p* < 0.001), only 22% initiated PrEP. Initiation was higher in female (82%, *p* < 0.001) and among the 15–19 years (51%, *p* = 0.11). PrEP uptake varied significantly by SDPs, lowest in clinics (9%, *p* < 0.001), higher in youth zones (58%, *p* < 0.001), and highest in schools (73%, *p* < 0.001). Adherence declined significantly over time, dropping from 12% at one month to 2% at six months (*p* < 0.001). These findings highlight the need for targeted interventions in schools and communities, addressing barriers such as awareness, side effects, and accessibility. Expanding PrEP services to non-traditional settings may enhance uptake and adherence, reducing HIV risk among young people.

**Supplementary Information:**

The online version contains supplementary material available at 10.1007/s10461-025-04719-6.

## Background

South Africa has an estimated 7.7 million people living with HIV (PLHIV) in 2023, representing a prevalence of 12.6% in the general population [1, 2]. Of these, 95% are aware of their status, 77% are receiving treatment, and among those on treatment, 71% have achieved viral suppression [2]. Young women aged 15–24 accounted for 28.7% of new HIV infections [1] and are about 3.6 times more likely to acquire HIV compared to their male counterparts. The difference is particularly notable among 10 to 19 year olds, with 24,000 adolescent girls becoming HIV-positive in 2023, compared to only 4,200 adolescent boys [1].

Adolescents and young women (AGYW) face increased vulnerability to HIV infection due to intergenerational sexual relationships, power imbalances in relationships, gender-based violence, limited economic opportunities, and inadequate access to secondary education [3, 4]. Poverty and gender power inequalities further exacerbate this vulnerability [5, 6]. Relationships with older men can create power imbalances, increasing the risk of intimate partner violence (IPV) and reducing the likelihood of knowing a partner’s HIV status or using condoms [4]. AGYW may also prioritize maintaining relationships, over long-term safety, like protecting themselves from HIV [3].

In response to the WHO recommendation for Pre-exposure prophylaxis (PrEP) for individuals at high risk [7], South Africa introduced PrEP in 2015 and began rolling it out in 2016 [8]. PrEP is an effective HIV prevention intervention for people at high-risk of HIV such as AGYW and adolescent boys and young men (ABYM) that offers several advantages, including autonomy and discretion in use at the time of sexual encounters [9, 10]. However, adherence challenges include lack of partner support, IPV, routine disruptions, and stigma, which can affect AGYW disproportionately [11–13]. Fear of partner reactions to PrEP use and concerns about concealment also contribute to adherence issues [14].

Current South African guidelines for PrEP target groups considered to be at high risk of HIV acquisition. These include sero-discordant couples where the HIV positive partner is not virally suppressed, AGYW, men who have sex with men, individuals with multiple sexual partners, people who inject drugs, those with a recent history of sexually transmitted infections (STIs), individuals who recognize their own risk and request PrEP, sex workers, and pregnant and breastfeeding women. Adolescents under 15 years of age are eligible if they have reached Tanner stage 3 or higher, indicating sexual maturity [15, 16].

Barriers to PrEP uptake include poor awareness, stigma, and inadequate service delivery [17, 18]. In South Africa, only 20% of university students are aware of PrEP, with higher awareness among those tested for HIV, primarily associated with adequate family support and discussions about HIV/STIs with sexual partners [15]. Adolescents and young adults often avoid sexual and reproductive health services due to stigma and poor service delivery [18–21].

Adherence to PrEP is still challenging, with many not recognising their risk factors, especially young women and men in heterosexual relationships often do not perceive that they are sufficiently at risk to warrant PrEP [22, 23]. Some believe that PrEP is harmful or dangerous, fear side effects and are concerned that they will be viewed as promiscuous or be suspected to be living with HIV [24]. Adherence to PrEP has several barriers, most of which affect AGYW more than other populations. Risk recognition not only affects women’s uptake of PrEP but also adherence. HIV uninfected women in sero-discordant relationships tend to be more adherent to PrEP, compared to those with partners of unknown status [14, 25–27].

Despite efforts, heterosexual men remain a crucial population in the fight against HIV. Compared to women, men are less likely to undergo HIV testing, seek timely care, and remain engaged in care [28–31]. Many men fear being perceived as HIV-positive if seen waiting at testing facilities, which deters them from seeking testing [32]. Unlike women, men often feel excluded from healthcare systems due to societal norms around masculinity [28]. Additionally, HIV stigma, homophobia, and traditional gender roles further hinder men’s participation in HIV prevention and treatment services [28].

To combat the high HIV incidence among AGYW and ABYM, South Africa has implemented various differentiated care models. These models offer tailored and streamlined HIV services to meet the specific needs of clients. One initiative is the Determined, Resilient, Empowered, AIDS-free, Mentored, and Safe (DREAMS) initiative. Launched by PEPFAR in 2014, DREAMS targets AGYW with a comprehensive strategy to identify HIV cases, link to treatment, and provide PrEP services to reduce HIV incidence among AGYW [33]. Other models, such as community- and school-based initiatives [31, 34, 35], offer a range of services including HIV counselling and testing, distribution of condoms, outreach teams and linkage officers to connect individuals to care. These models also incorporate programmes addressing sexual assaults and gender-based violence [36], initiatives targeting men [37, 38], services operating outside regular working hours, and the provision of PrEP [39].

Despite various PrEP-delivery approaches to improve PrEP uptake and adherence in South Africa, there remains a lack of understanding regarding the optimization of PrEP uptake among AGYW and ABYM. These groups are particularly vulnerable to HIV infection due to a combination of biological, social, and structural factors [40]. The high HIV incidence among AGYW and ABYM highlights the urgent need for effective PrEP interventions tailored to their unique needs [41].

Additionally, there is limited evidence comparing different intervention models aimed at improving oral PrEP uptake and adherence for these populations. While facility-based models, which deliver PrEP services through health clinics, are the most common, they often face barriers such as long wait times, stigma, and transportation challenges [42]. School-based models aim to integrate PrEP services within educational settings, potentially increasing accessibility for students but may encounter issues related to parental consent and school policies [43]. Community-based models involve delivering PrEP services within community settings, leveraging local organizations and networks to reach AGYW and ABYM more effectively but may struggle with consistency and resource allocation [44]. Hybrid models combine elements of these approaches to maximize reach and efficiency, yet their effectiveness and feasibility require further investigation [14].

This study examined various service delivery models (facility-based, school-based, community-based, and hybrid models) and their relationship to key PrEP indicators such as initiation, adherence, and retention in care rates. By comparing these models, the study aimed to identify effective and feasible oral PrEP delivery strategies tailored to enhance uptake, and adherence among at-risk AGYW and ABYM in the uMgungundlovu district, KwaZulu-Natal province, South Africa.

## Methods

### Study Design and Setting

We conducted a longitudinal, population-based cohort study at 22 purposively selected oral PrEP service delivery points (SDPs) i.e., clinics, schools, and community-based youth zones, across the seven subdistricts of the uMgungundlovu district is KwaZulu-Natal. Newly diagnosed HIV-negative, sexually active, high-risk AGYW (15–24 years) and ABYM (15–35 years) were recruited over a 12-month period, from August 2021 to July 2022. Participants were followed up at one, four, and seven months after initiating oral PrEP. In addition, routine service records were assessed at enrolment and at 1, 4, and 7 months to obtain HIV test results, and to describe PrEP initiation and continuation. A detailed description of the study design and settings have been published previously elsewhere [45].

### Conceptual Framework

**Fig. 1:**
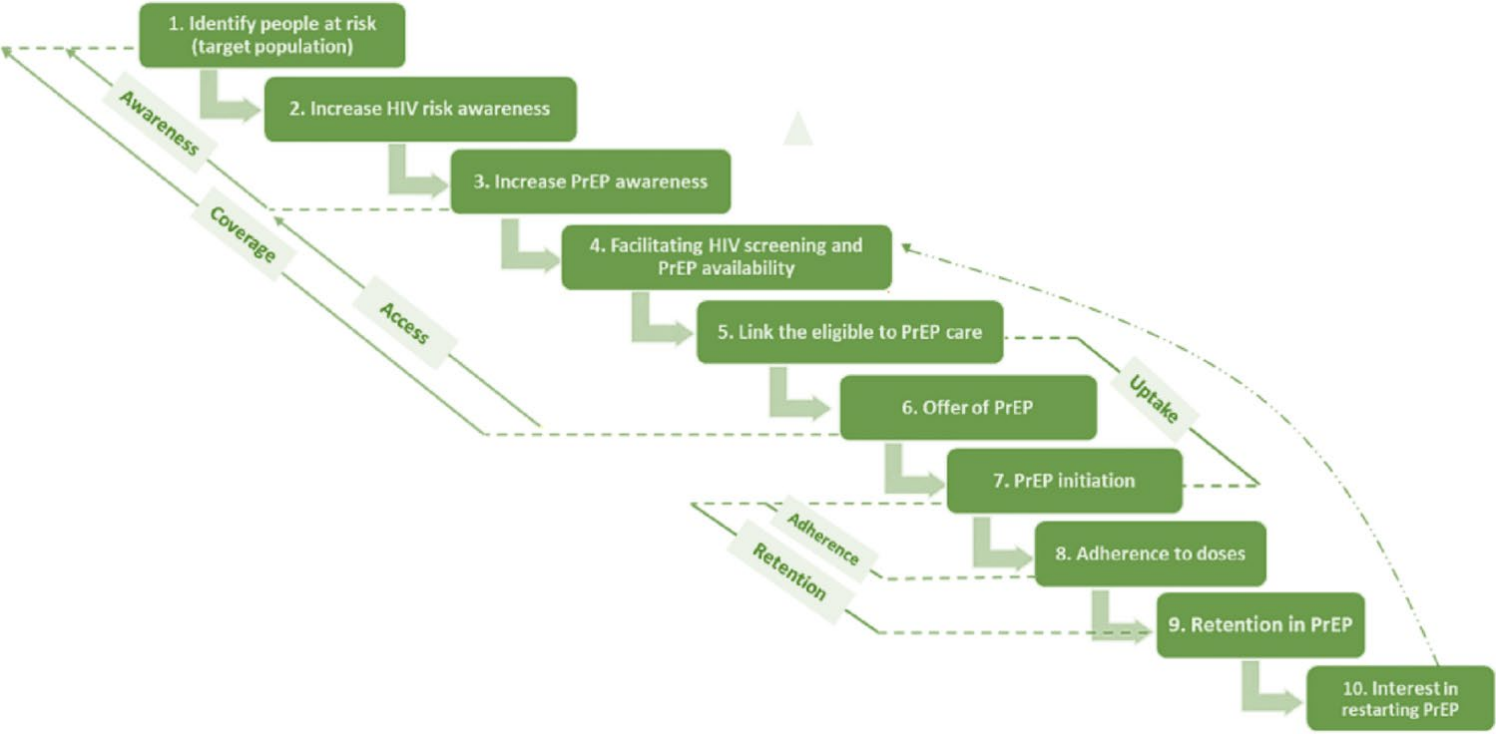
PrEP initiation and adherence continuum. Adapted from Dunbar et al. [7]

This study was guided by the PrEP initiation and adherence continuum (Fig. 1), which is crucial for assessing the progress and effectiveness of implementing PrEP programmes. The continuum encompasses five broad categories: awareness, access, uptake, adherence, and continuation, each comprising different steps.

PrEP **awareness** involves three steps, (i) identifying individuals at highest risk for contracting HIV, (ii) increasing HIV risk awareness education among those individuals, and (iii) enhancing PrEP awareness. PrEP **access** also involves three steps, (iv) facilitating HIV screening and PrEP availability, (v) linking to PrEP care, and (vi) offering PrEP. **Uptake** involves (vii) initiating PrEP, while **adherence** involves (viii) measuring individual adherence to PrEP. Finally, **continuation** involves (ix) retaining individuals in PrEP care [46]. Each of these steps presents opportunities for patients to either continue using PrEP or disengage from it. This paper focuses on two of the broad categories in the continuum, PrEP uptake and adherence.

### Sample Size Calculation

#### Selection of Participating Service Delivery Points (SDPs)

Excluding correctional facilities and non-medical sites, a total of 64 public sector facilities in uMgungundlovu district provide antiretroviral therapy (ART) services. These include 46 fixed clinics, 17 mobile clinics, and one state-aided clinic. To ensure feasibility in reaching the required enrolment, only facilities that reported over 4,000 HIV test per month and an average of at least 10 new HIV-positive cases per month (based on District Health Information System data from September 2019 to August 2020) were considered.

The final selection of 22 SDPs for this study was based on the presence of an ongoing intervention (the DREAMS core package of interventions [33]) targeting adolescent girls and young women (AGYW), as well as the linkage between primary health care facilities and key community sites, These sites include Technical and Vocational Education and Training (TVET) colleges, high schools, community youth zones, and men-linked services. Within each of the seven strata/sub-districts, SDPs were selected based on convenience while ensuring alignment with these criteria.

#### Sample Size for Individuals to be Enrolled

Preliminary findings from a sub-study nested within a clinical trial measuring HIV incidence in Durban, South Africa, estimated a 46% rate of PrEP initiation [47]. Given the similarity in populations, we applied this 46% initiation rate to uMgungundlovu. To account for potential variation, we also considered initiation rates ranging from 21 to 46%, based on estimates from comparable populations. The design entails a non-randomised comparison of four units in each service delivery type as outlined in Table 1. Sample size calculations were designed to assess the study’s power to detect differences in coverage across service delivery models. These calculations compare proportions between clusters of fixed size in each group at a specific time point (i.e., 6 months). The sample size and power were computed using Stata v18.0.

**Table 1:** Power and sample size for the comparison of service delivery model

Models	Alpha	Beta	k_1_	k_2_	m_1_	m_2_	Delta	*p*_1_	*p*_2_	Rho
Community vs. Facility models	0.05	0.80	4	4	50	50	0.25	0.21	0.46	0.04
Community vs. School models	0.05	0.80	4	4	20	20	0.25	0.21	0.46	0.02

Where alpha—type 1 error; beta—power; m—average cluster size; k—number of clusters; delta—difference between proportions; p—PrEP initiation estimate (proportion); rho—inter-cluster correlation

A sample size of 200 per service delivery model, was deemed sufficient for this study, with an additional 50 participants allocated to account for potential dropouts and non-responses, resulting in a total minimum sample of 650. For detailed sampled PrEP service delivery points, refer to [45].

### Data Collection Procedures

**Fig. 2:**
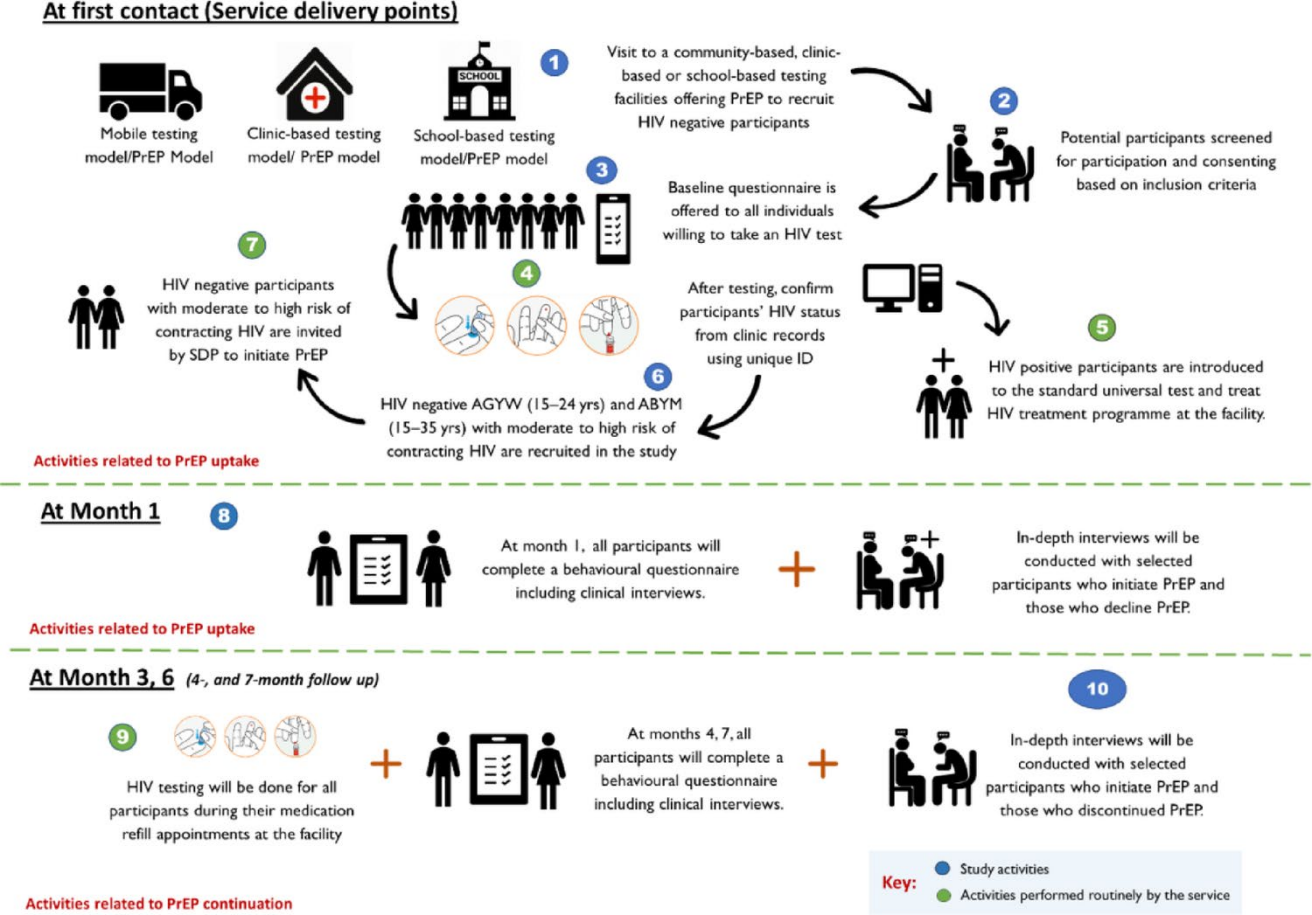
An illustration of the study activities

This study followed the steps illustrated in Fig. 2. Baseline quantitative data collection was conducted using self-administered electronic questionnaires built into Research electronic data capture (REDCap) [48]. Questionnaires were administered to eligible participants by trained fieldworkers at baseline and at 1-, 4- and 7-month follow-up visits to assess factors influencing engagement with oral PrEP. A 28-day window was allowed for PrEP refills and clinical records updates in the TIER.Net, an electronic database for Infectious Disease Epidemiology and Research system [49]. Clinical follow-ups occurred at 1-, 3-, and 6-month, enabling healthcare providers to assess participants’ eligibility to continue PrEP, as outlined in the PrEP Standard Operating Procedure (SOP). During this exercise, socio-demographic information was collected including age, sex, employment status and level of education attained. We also collected behavioural and HIV risk-related data, including the number of sexual partners, partner HIV status, condom use, recreational drug use, alcohol use and post-exposure prophylaxis.

To be eligible for the study, participants had to meet the following criteria: they must have accepted an HIV test at a participating SDPs between August 2021 and January 2022, had access to a cell phone, and provided contact details. Additionally, participants had to be within the age range of 15–24 years for AGYW or 15–35 years for ABYM, self-reported as sexually active, be identified as high risk for HIV infection through a risk assessment, test seronegative on the day of recruitment, and were able and willing to provide written informed consent.

### Screening and Recruitment

Participants were invited to enrol while waiting in the queue for HIV counselling at selected SDPs. This approach prevented immediate recruitment after receiving a positive HIV test result, thereby addressing ethical concerns. Individuals willing to participate received study information, underwent screening for eligibility in a private room, and provided informed consent, granting researchers access to their HIV test results, healthcare records, and track health outcomes.

AGYW and ABYM who tested HIV negative were offered a Rapid Risk Assessment Screening Tool (Appendix 1) by the service delivery staff. The tool assessed PrEP eligibility across various domains, including unprotected sex, having multiple sexual partners, engaging in sex with a partner of an unknown or positive HIV status, and engaging in sexual activity under the influence of drugs. Additional factors assessed included engaging in sexual activity for favours, a recent history of STIs, intravenous drug use, and being in a sero-discordant relationship where the HIV-positive partner was not virally suppressed, in accordance with the South African National department of Health (NDOH) guidelines.

PrEP risk was defined using the risk screening scores adapted from [50] and categorised as low (0–6), moderate (7–25), or high (26+), based on participants’ responses to the questions in the screening tools. Responses included nominal options such as “don’t know” and “prefer not to answer,” allowing for more nuanced feedback by capturing risk even when participants chose not to respond to specific questions. Participants identified as being at substantial risk upon considering the different domains were considered eligible for oral PrEP and were offered PrEP by the staff at the service delivery points (Fig. 2). Participants were at substantial risk if they met any of the following criteria: (i) had multiple sexual partners, (ii) had a positive STI result in the past 6 months, (iii) had an HIV-positive partner, (iv) inconsistent condom use, and (v) did not know their partner’s HIV status.

### Data Analysis

At baseline, demographic and behavioural characteristics were analysed, stratified by service delivery models (SDPs). Categorical variables were analysed using proportions and percentages with their respective 95% confidence intervals. Continuous data were presented as means (± SD) or medians (IQR) depending on data distribution. The association between demographic/behavioural characteristics, PrEP initiation and adherence, stratified by SDP-types were assessed using Pearson’s Chi-square/Fisher’s exact tests and a binomial regression model with a robust variance option. Service delivery models were compared based on gender and age, although it was anticipated that PrEP initiation and continuation would occur at hybrid sites rather than exclusively at facility-only or community-only locations. Hence, a descriptive analysis was performed, with attempts made to establish statistical associations between adherence and different SDPs whenever feasible.

Further analysis on the sociodemographic characteristics of individuals who initiated PrEP was conducted during follow-up assessments at 1 (t1), 4 (t3), and 7 (t6) months outlined in the PrEP SOP. PrEP uptake and continuation were reported as the proportion of adherent participants relative to the baseline sample of HIV-negative individuals or those who initiated PrEP, respectively. Adherence was defined as taking four or more daily pills in the previous seven days, based on patient self-reports at each clinical follow-up visit.

Given the limited number of clusters, the comparison of SDPs was conducted at the cluster level, as analysing at the participant level was not recommended. Due to the exploratory nature of the study design, the descriptive analysis of coverage profiles over time was emphasised as valuable information from the statistical analysis. The three repeated measures in each participant provided the study with the opportunity to capture adherence over time and assess the potential facilitators and barriers enhancing adherence. Statistical significance was determined with a p-value of < 0.05 for all tests.

### Ethics Consideration

Ethical approval for this study was obtained from the South African Medical Research Council’s Health Research Ethics Committee (Ref #: EC051 - 11/2020) on 19 January 2021. Permissions were also obtained from the KwaZulu-Natal Provincial Department of Health (Ref #: KZ_202010_033), the uMgungundlovu health districts, and facilities. For participants aged 15–17 years, a waiver of parental consent was approved, though the informed consent process and documentation were identical to those for participants 18 years and older. Additionally, both oral and written informed consent were obtained from all potential participants in the study prior to their participation.

## Results

**Fig. 3:**
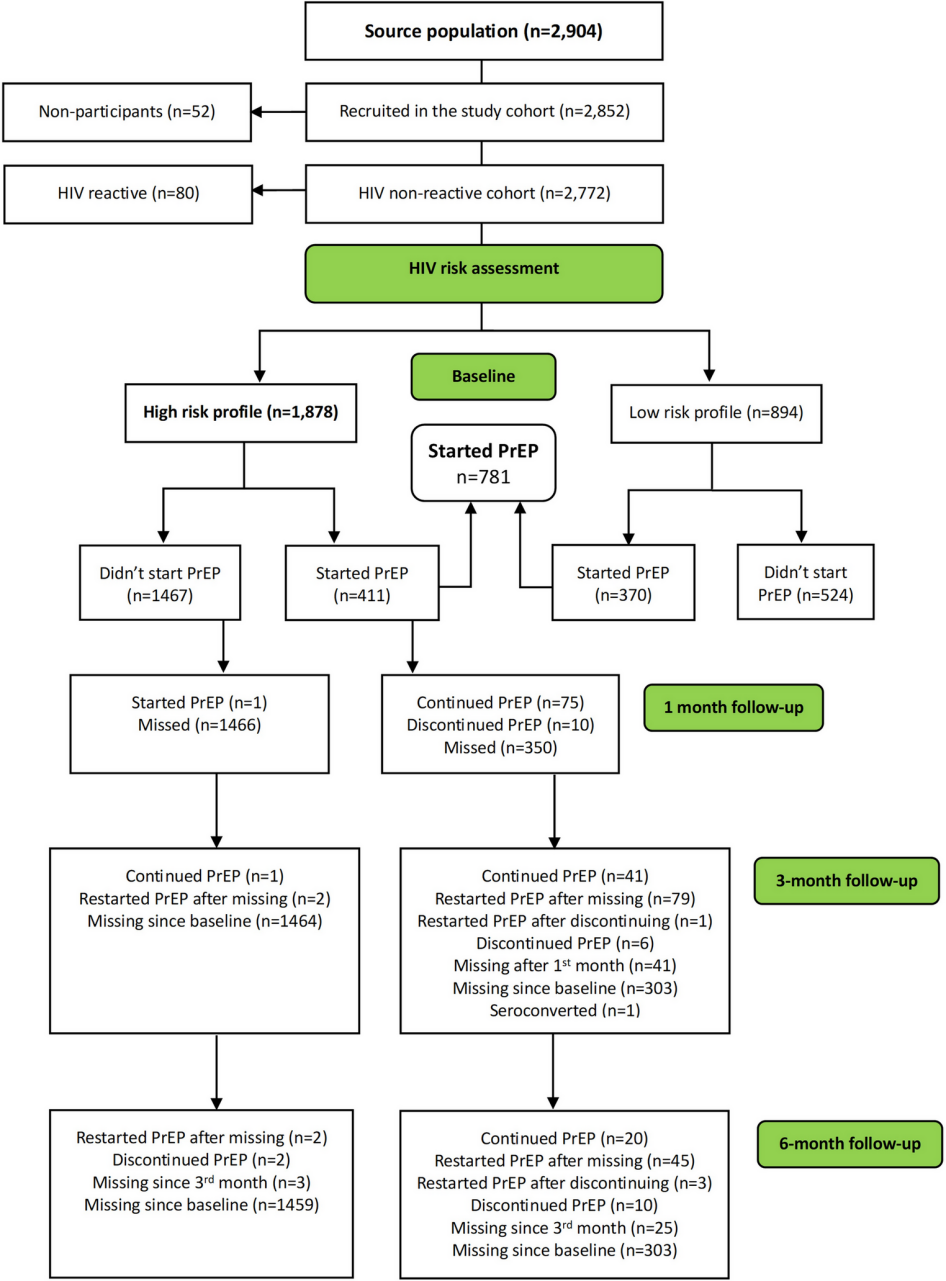
Baseline sample consort diagram detailing the recruitment of study participants into the PrEP study in uMgungundlovu district between August 2021 to July 2022

Between August 2021 and July 2022, a total of 2,852 participants were recruited from 22 selected PrEP SDPs after screening 2,904 individuals across seven sub-districts in the uMgungundlovu district (Fig. 3). Most participants were recruited from clinics (66%, 1,833/2,772), while the Youth Zones and schools accounted for 17% (472/2,772) and 16.8% (467/2,772) respectively (Table 3). Of the 2,852 participants recruited, 80 were HIV-positive, 894 had a low HIV risk profile for PrEP based on the risk assessment, and 1,878 were classified as high risk. Overall, 781 participants initiated PrEP regardless of their HIV risk profile, including 411 classified as high risk.

**Fig. 4:**
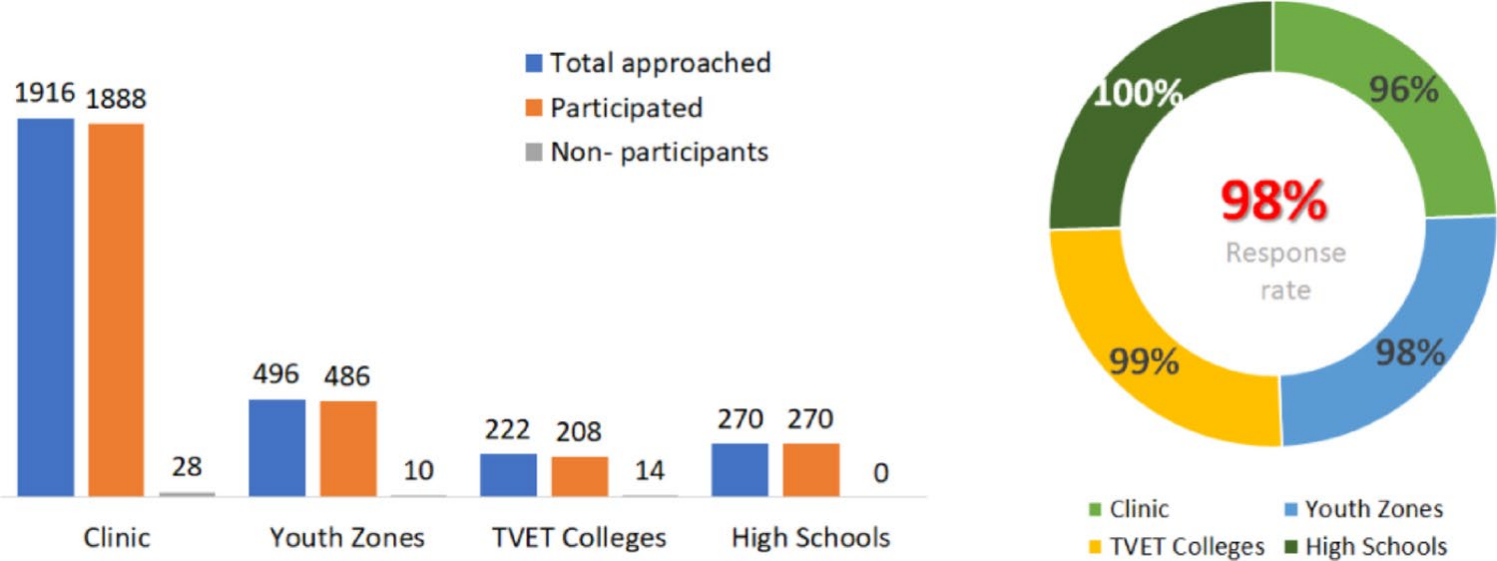
Response rate showing overall proportion against the target by service delivery points (*n* = 2,904)

Figure 4 provides an overview of the total responses by SDPs. Response rates were calculated for each SDPs based on the number of participants approached. The overall response rate of 98% (2852/2904) indicates that the results are representative of the target population. For this analysis, participants were grouped into three SDPs: Clinics, Youth zones and Schools (including TVET colleges and high schools) as shown in Table 2.

**Table 2:** Socio-demographic characteristics of AGYW and ABYM at baseline by SDPs in uMgungundlovu district between August 2021 to July 2022

Variable	Total (N1 = 2772)		Clinics (N2 = 1833)		Youth Zone (N3 = 472)		Schools (N4 = 467)			
Demographic variables	n	% (95% CI)	n	% (95% CI)	n	% (95% CI)	n	% (95% CI)	F-value	Pearson chi-square p-value
**PrEP initiation**										
Initiated PrEP	781	28.2 (17.5–42.1)	165	9.0 (4.4–17.5)	275	58.3 (36.5–77.3)	341	73.0 (58.2–84.0)	33.1	**< 0.001**
Did not initiate PrEP	1991	71.8 (57.9–82.5)	1668	91.0 (82.6–95.6)	197	41.7 (22.7–63.5)	126	27.0 (16.0–41.8)		
**Nationality**										
South African	2751	99.2 (98.3–99.7)	1816	99.1 (97.7–99.6)	740	99.6 (95.9–100)	465	99.6 (97.4–99.9)	0.1	0.90
Other SADC	18	0.6 (0.3–1.4)	15	0.8 (0.3–2.0)	1	0.2 (0.0–2.1)	2	0.4 (0.1–2.6)		
Other African	2	0.1 (0.0–0.2)	2	0.1 (0.0–0.4)	0	0	0	0		
Outside Africa	1	0.0 (0.0–0.3)	0	0	1	0.2 (0.0–2.1)	0	0		
**Ethnicity**										
**N**_**1**_ **= 2768; N**_**2**_** = 1831; N**_**3**_** = 472; N**_**4**_** = 467**										
Black African	2748	99.3 (98.0–99.7)	1814	99.1 (97.2–99.7)	469	99.8 (97.9–100)	465	99.6 (97.4–99.9)	0.1	0.89
Coloured	14	0.5 (0.2–1.3)	12	0.7 (0.2–1.8)	1	0.2 (0.0–2.1)	1	0.2 (0.0–1.3)		
White	2	0.1 (0.0–0.5)	2	0.1 (0.0–0.6)	0	0	0	0		
Indian/Asian	2	0.1 (0.0–0.2)	2	0.1 (0.0–0.4)	0	0	0	0		
Other	1	0.0 (0.0–0.3)	0	0	0	0	1	0.3 (0.0–1.3)		
**Median Age (years)**	2772	21 (19–24)	1833	22 (20–24)	472	20 (17–22)	467	18 (16–21)	435	**< 0.001**
**Sex**										
Male	1078	38.9 (30.1–48.5)	850	46.4 (35.1–58.0)	92	19.5 (14.2–26.1)	136	29.1 (23.8–35.1)	14.7	**< 0.001**
Female	1694	61.1 (51.5–69.9)	983	53.6 (42.0–64.9)	380	80.5 (73.9–85.78)	331	70.9 (64.9–76.2)		
**Age categories**										
15–19 years	857	30.9 (19.4–45.4)	373	20.4 (13.7–29.2)	221	46.8 (36.3–57.7)	263	56.3 (9.9–93.8)	2.8	0.11
20–24 years	1384	49.9 (40.3–59.6)	982	53.6 (49.6–57.5)	217	46.0 (36.0–56.3)	185	39.6 (6.6–86.0)		
25–29 years	337	12.2 (8.8–16.5)	292	15.9 (12.0–20.9)	28	5.9 (4.1–8.5)	17	3.6 (0.6–18.8)		
30–35 years	194	7.0 (4.2–11.4)	186	10.2 (6.5–15.5)	6	1.3 (0.4–3.6)	2	0.4 (0.1–2.7)		
**Highest education attained**										
**N**_**1**_ **= 2769; N**_**2**_ **= 1833; N**_**3**_ **= 471; N**_**4**_** = 4655**										
No education	2	0.1 (0.0–0.3)	2	0.1 (0.0–0.4)	0	0	0	0	0.9	0.41
Primary education	33	1.2 (0.9–1.6)	21	1.1 (0.8–1.7)	12	2.5 (2.2–3.0)	0	0		
High school education	2533	91.5 (84.0–95.6)	1639	89.4 (79.2–95.0)	443	94.1 (91.5–95.9)	451	97.0 (84.5–99.5)		
Tertiary	196	7.1 (3.2–15.0)	167	9.1 (3.9–20.0)	15	3.2 (1.8–5.6)	14	3.0 (0.5–15.5)		
**Currently studying**										
**N**_**1**_ **= 2766; N**_**2**_** = 1828; N**_**3**_** = 471; N**_**4**_** = 467**										
No	1241	44.9 (36.7–53.3)	1055	57.7 (47.3–67.5)	181	38.4 (28.3–49.7)	5	1.1 (0.2–5.0)	48.3	**< 0.001**
Yes	1523	55.1 (46.7–63.2)	771	42.2 (32.6–52.4)	290	61.6 (50.3–71.7)	462	98.9 (95.0–99.8)		
**Marital status**										
**N**_**1**_ **= 2763; N**_**2**_ **= 1825; N**_**3**_ **= 472; N**_**4**_ **= 466**										
Married (living together)	19	0.7 (0.3–1.5)	18	1.0 (0.4–2.3)	1	0.2 (0.0–2.1)	0	0	2.1	0.14
Married (living separately)	9	0.3 (0.1–0.8)	4	0.2 (0.0–1.2)	0	0	5	1.1 (0.5–2.1)		
Cohabiting	103	3.7 (2.5–5.6)	90	4.9 (3.1–7.7)	6	1.3 (0.5–3.1)	7	1.5 (0.2–9.3)		
Dating (living separately)	1947	70.5 (65.7–74.9)	1324	72.6 (68.2–76.5)	334	70.8 (67.1–74.2)	289	62.0 (40.9–79.4)		
Single	66	24.1 (19.5–29.5)	379	20.8 (16.6–25.7)	125	26.5 (23.2–30.1)	162	34.8 (16.4–59.1)		
**Socio-economic variables**										
**Worked past 12 months**										
**N**_**1**_ **= 2754; N**_**2**_ **= 1823; N**_**3**_ **= 465; N**_**4**_ **= 466**										
Never worked	1607	58.4 (51.4–65.0)	985	54.0 (43.5–64.2)	279	60.0 (54.7–65.1)	343	73.6 (69.3–77.5)	8.6	**< 0.001**
Once in a while	262	9.5 (5.5–16.1)	242	13.3 (7.9–21.5)	12	2.6 (2.2–3.0)	8	1.7 (1.4–2.2)		
Most months	263	9.6 (7.5–12.1)	215	11.8 (9.0–15.4)	33	7.1 (4.8–10.4)	15	3.2 (1.4–7.3)		
Every month	585	21.2 (15.5–28.4)	360	19.8 (12.2–30.4)	133	28.6 (26.0–31.4)	92	19.7 (15.4–24.9)		
Don’t know	25	0.9 (0.7–1.2)	14	0.8 (0.5–1.1)	5	1.1 (0.8–1.5)	6	1.3 (1.0–1.7)		
Prefer not to answer	12	0.4 (0.2–0.9)	7	0.4 (0.1–1.2)	3	0.6	2	0.4 (0.1–2.7		
**Food insecurity**										
Low	2263	81.6 (75.5–86.5)	1488	81.2 (72.0–88.9)	381	80.7 (72.0–87.9)	394	84.4 (70.9–92.3)	0.9	0.41
Medium	359	13.0 (9.0–18.2)	255	13.9 (8.5–21.9)	54	11.4 (9.6–13.6)	50	10.7 (4.5–23.5)		
High	53	1.9 (1.1–3.2)	31	1.7 (0.7–3.9)	17	3.6 (2.7–4.8)	5	1.1 (0.5–2.1)		
Prefer not to answer	97	3.5 (2.9–4.2)	59	3.2 (2.6–4.0)	20	4.2 (3.0–6.0)	18	3.9 (2.6–5.7)		
**Household income**										
**N**_**1**_ **= 2765; N**_**2**_ **= 1823; N**_**3**_ **= 465; N**_**4**_ **= 466**										
R0–999	1947	70.4 (61.2–78.2)	1181	64.5 (52.6–74.9)	397	84.7 (80.8–87.9)	369	79.2 (75.1–82.7)	12.9	**< 0.001**
R1000–4999	418	15.1 (9.9–22.4)	357	19.5 (12.8–28.6)	38	8.1 (7.3–9.0)	23	4.9 (2.2–10.8)		
R5000+	118	4.3 (2.1–8.5)	113	6.2 (3.1–12.0)	3	0.6 (0.2–1.9)	2	0.4 (0.1–2.1)		
Don’t know	179	6.5 (5.0–8.4)	110	6.0 (4.3–8.3)	14	3.0 (2.1–4.2)	55	11.8 (8.9–15.4)		
Prefer not to answer	103	3.7 (2.7–5.1)	69	3.8 (2.5–5.8)	17	3.6 (2.2–5.8)	17	3.6 (2.3–5.8)		
**Received child grant**										
**N**_**1**_ **= 2767; N**_**2**_ **= 1830; N**_**3**_ **= 470; N**_**4**_ **= 467**										
No	923	33.4 (26.1–41.5)	699	38.2 (29.0–48.3)	103	21.9 (19.9–24.1)	121	25.9 (18.9–34.4)	5.4	**< 0.01**
Yes	1752	63.3 (55.1–70.8)	1067	58.3 (48.1–67.8)	362	77.0 (75.3–78.6)	323	69.2 (64.0–73.9)		
Don’t know	85	3.1 (2.1–4.5)	58	3.2 (2.0–5.0)	5	1.1 (0.4–2.6)	22	4.7 (2.1–10.2)		
Prefer not to answer	7	0.3 (0.1–0.7)	6	0.3 (0.1–1.0)	0	0	1	0.2 (0.0–1.4)		
**Received disability grant**										
**N**_**1**_ **= 2706; N**_**2**_ **= 1793; N**_**3**_ **= 468; N**_**4**_ **= 445**										
No	2419	89.4 (86.4–91.8)	1634	91.1 (88.0–93.5)	397	84.8 (77.0–90.3)	388	87.2 (84.7–89.4)	4.0	**0.04**
Yes	287	10.6 (8.2–13.6)	159	8.9 (6.5–12.0)	71	15.2 (9.7–23.0)	57	12.8 (10.6–15.3)		
**Received social grant**										
**N**_**1**_ **= 2767; N**_**2**_ **= 1829; N**_**3**_ **= 472; N**_**4**_ **= 466**										
No	1511	54.6 (50.4–58.7)	1043	57.0 (51.1–62.8)	240	50.9 (44.6–57.1)	228	48.9 (40.7–57.2)	2.0	0.14
Yes	1148	41.5 (38.2–44.8)	707	38.7 (33.9–43.7)	227	48.1 (41.5–54.8)	214	45.9 (43.2–48.6)		
Don’t know	104	3.8 (2.3–6.1)	76	4.2 (2.5–6.8)	4	0.8 (0.3–2.1)	24	5.2 (1.3–17.8)		
Prefer not to answer	4	0.1 (0.0–0.6)	3	0.2 (0.0–0.9)	1	0.2 (0.0–1.5)	0	0		
**Sexual experience and other related factors**										
**Age at sexual debut**										
**N**_**1**_ **= 2,206; N**_**2**_ **= 1626; N**_**3**_ **= 323; N**_**4**_ **= 257**										
≤ 14 years	149	6.8 (4.9–9.2)	121	7.4 (5.6–9.8)	7	2.2 (1.3–3.7)	21	8.2 (1.7–31.1)	0.8	0.44
15–17 years	1009	45.7 (40.3–51.2)	702	43.2 (37.5–49.1)	177	54.8 (47.1–62.3)	130	50.6 (29.8–71.2)		
≥ 18 years	987	44.7 (39.4–50.2)	746	45.9 (40.8–51.1)	136	42.1 (34.9–49.7)	105	40.9 (14.7–73.5)		
Don’t know	42	1.9 (1.0–3.8)	40	2.5 (1.3–4.7)	2	0.6 (0.1–6.0)	0	0		
Prefer not to answer	19	0.9 (0.4–1.7)	17	1.0 (0.5–2.1)	1	0.3 (0.0–2.3)	1	0.4 (0.1–1.4)		
**First sex experience**										
**N**_**1**_ **= 2206; N**_**2**_ **= 1624; N**_**3**_ **= 324; N**_**4**_ **= 258**										
Wanted	1766	80.1 (75.4–84.0)	1332	82.0 (76.3–86.6)	235	72.5 (58.3–83.3)	199	77.1 (67.7–84.4)	1.5	0.23
Persuaded	388	17.6 (13.6–22.5)	258	15.9 (11.1–22.2)	79	24.4 (14.7–37.7)	51	19.8 (13.9–27.4)		
Raped	39	1.8 (1.2–2.5)	28	1.7 (1.2–2.6)	6	1.9 (0.6–5.6)	5	1.9 (0.7–5.3)		
Don’t know	3	0.1 (0.0–0.5)	2	0.1 (0.0–0.5)	0	0	1	0.4 (0.0–5.5)		
Prefer not to answer	10	0.5 (0.2–0.9)	4	0.2 (0.1–0.8)	4	1.2 (0.4–3.5)	2	0.8 (0.2–2.7)		
**Lifetime sexual partners**										
**N**_**1**_ **= 2201; N**_**2**_ **= 1622; N**_**3**_ **= 322; N**_**4**_ **= 257**										
One partner	469	21.3 (17.5–25.7)	295	18.2 (14.3–22.9)	90	28.0 (19.8–37.8)	84	32.7 (20.0–48.6)	4.6	**< 0.01**
Two–three partners	746	33.9 (29.4–38.7)	516	31.8 (26.7–37.5)	133	41.3 (34.7–48.3)	97	37.7 (35.3–40.3)		
Four or more partners	843	38.3 (33.2–43.6)	685	42.2 (36.7–48.0)	87	27.0 (18.2–38.1)	71	27.6 (17.9–40.0)		
Don’t know	113	5.1 (3.1–8.4)	101	6.2 (3.8–10.0)	10	3.1 (1.1–8.3)	2	0.8 (0.2–2.9)		
Prefer not to answer	30	1.4 (0.7–2.6)	25	1.5 (0.8–3.1)	2	0.6 (0.2–2.2)	3	1.2 (0.4–3.1)		
**Sex with main partner**										
**N**_**1**_ **= 2182; N**_**2**_ **= 1614; N**_**3**_ **= 318; N**_**4**_ **= 250**										
No	242	11.1 (6.9–17.3)	204	12.6 (7.6–20.4)	17	5.3 (3.6–7.8)	21	8.4 (5.8–12.0)	2.4	0.10
Yes	1905	87.3 (81.6–91.4)	1381	85.6 (78.6–90.5)	299	94.0 (91.9–95.6)	225	90.0 (85.1–93.4)		
Prefer not to answer	35	1.6 (1.0–2.5)	29	1.8 (1.2–2.7)	2	0.6 (0.2–2.1)	4	1.6 (0.2–14.8)		
**Sex with additional partner**										
**N**_**1**_ **= 2179; N**_**2**_ **= 1613; N**_**3**_ **= 317; N**_**4**_ **= 249**										
No	1434	65.8 (60.3–71.0)	1016	63.0 (55.7–69.8)	230	72.6 (65.5–78.7)	188	75.5 (68.6–81.3)	3.5	**0.02**
Yes	729	33.5 (28.2–39.1)	585	36.3 (29.4–43.8)	85	26.8 (21.2–33.3)	59	23.7 (17.6–31.1)		
Prefer not to answer	16	0.7 (0.4–1.3)	12	0.7 (0.4–1.4)	2	0.6 (0.1–4.5)	2	0.8 (0.2–2.8)		
**On contraceptives**										
**N**_**1**_ **= 1690; N**_**2**_ **= 987; N**_**3**_ **= 379; N**_**4**_** = 324**										
No	974	57.6 (50.9–64.1)	494	50.1 (41.2–58.9)	240	63.3 (57.2–69.0)	240	74.1 (53.0–87.9)	4.4	**0.03**
Yes	709	42.0 (35.7–48.5)	489	49.5 (41.0–58.1)	137	36.2 (31.0–41.6)	83	25.6 (12.2–46.1)		
Prefer not to answer	7	0.4 (0.2–0.9)	4	0.4 (0.1–1.2)	2	0.5 (0.2–1.8)	1	0.3 (0.0–2.1)		

The p-value of 0.05 was considered statistically significant is given in bold

p-values derived using Person Chi-squared test considered the one stage cluster design

Proportions (%) for the columns reported as n/N (except if data is missing - denominator added in the n column) and the associated 95% CI

*CI* Confidence interval

Table 2 presents the socio-demographic and behavioural characteristics of the participants at baseline, categorised by SDPs. It indicates that most of the participants were Black African (99.2%). Approximately two-thirds (66%, 1833/2772) were recruited from clinics, while schools (TVET colleges and high schools) accounted for the lowest proportion of recruits at 17% (467/2772. Despite high recruitment rates, PrEP initiation was significantly low among clinics (9%, 165/1,833), higher in youth zones (58%, 275/472) and highest in schools (73%, 341/467); (*p* < 0.001). Among the 2,772 participants reported as HIV non-reactive on testing, 1,694 (61%) were females (*p* < 0.001), 1,384 (50%) were aged between 20 and 24 years (*p* = 0.11), and 1,947 (71%) were in dating relationships (living separately) (*p* = 0.14). More than half (55%, 1,523/2,766) of the participants were currently enrolled in school (*p* = 0.41), and 70% reported having a monthly household income of less than R1,000 (US$54).

### PrEP Initiation Rates Among AGYW and ABYM

**Fig. 5:**
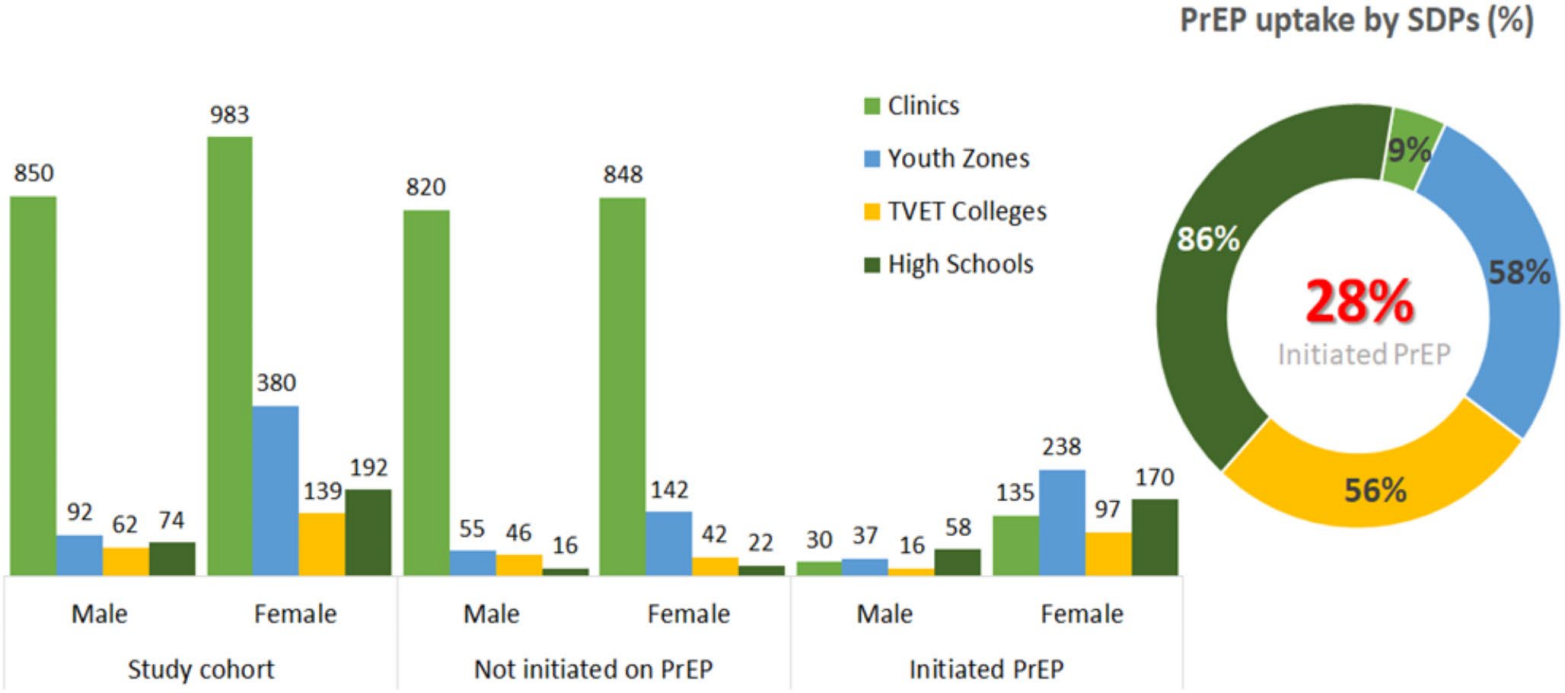
PrEP initiation rates (*n* = 2,772) by service delivery models and gender, KwaZulu-Natal, South Africa, 2022

The PrEP initiation rate was relatively low, with above a quarter of those HIV non-reactive (28%, 781/2,772) initiating (Fig. 5; Table 3). Despite two-thirds (65%, 1,878/2,772) of the participants having a high-risk profile for HIV infection based on the risk assessment screening tool and being eligible for PrEP, only 22% (411/1,878; *p* < 0.001) went on to initiate PrEP (Fig. 3; Table 3).

**Table 3:** PrEP initiation rates (*n* = 2772) by service delivery models and HIV infection risk profile, KwaZulu-Natal, South Africa, 2022

Service delivery model	High risk profile, *n* (%)			Low risk profile, *n* (%)			Total, *n* (%)
	Not initiated on PrEP	Initiated on PrEP	Pearson chi-square *p*-value	Not initiated on PrEP	Initiated on PrEP	Pearson chi-square *p*-value	
Clinics	1270 (91)	118 (9)	**< 0.001**	398 (89)	47 (11)	**< 0.001**	1833 (9)
Youth zones	119 (44)	154 (56)		78 (39)	121 (61)		472 (58)
TVET colleges	61 (43)	81 (57)		27 (46)	32 (54)		201 (56)
High schools	17 (23)	58 (77)		21 (11)	170 (89)		266 (86)
Total	1467 (78)	411 (22)		524 (59)	370 (41)		2772 (28)

The p-value of 0.05 was considered statistically significant is given in bold

Figure 5 indicates that among those who initiated PrEP, approximately 86% (228/266) were from high schools, compared to community-based youth zones (58%, 275/472), TVET colleges (56%, 113/201), with the lowest initiation rates observed at the healthcare facilities/clinics (9%, 165/1,833).

Table 3 shows that participants who initiated PrEP while classified as low risk for HIV infection comprised 41% (370/894) of the total, with most initiations coming from the high schools’ service delivery model (89%, 170/191; *p* < 0.001). Significant differences in PrEP initiation rates were observed across various types of SDPs for those classified as high risk for HIV infection. The high school service delivery model had the highest proportion of initiations (77%, 58/75), followed by TVET colleges (57%, 81/142) and Youth Zones (56%, 154/273), and facilities/clinics had the least initiations (9%, 118/1,388).

Overall, there was a noticeable gender disparity (Fig. 5), with higher initiation rates among female participants from high schools (89%, 170/192), TVET colleges (70%, 97/139), Youth Zones (63%, 238/380), and facilities/clinics (14%, 135/983). A similar trend in PrEP initiation rates was also observed among male participants: high schools (78%, 58/74), youth zones (40%, 37/92), TVET colleges (26%, 16/62) and facilities/clinics (4%, 30/850).

Among participants classified as high risk for HIV infection, the gender disparity remains consistent. Female participants had the highest initiation rates across all SDPs, with rates of 81% (39/48) in high schools, 72% (67/93) in TVET colleges, 61% (127/208) in Youth Zones and 14% (91/669) in facilities/clinics. Male participants followed a similar trend, with PrEP initiation rates of 70% (19/27) in high schools, 42% (27/65) in youth zones, 29% (14/49) in TVET colleges and 4% (27/719) in facilities/clinics (Table 4).

**Table 4:** PrEP initiation rates among the high-risk profile participants (*n* = 1878) by gender, SDPs, and age categories in KwaZulu-Natal, South Africa, 2022

Variables	Males, *n* (%)			Females, *n* (%)			Total, *n* (%)
	Not initiated on PrEP, 773 (90)	Initiated on PrEP, 87 (10)	Pearson chi-square *p*-value	Not initiated on PrEP, 694 (68)	Initiated on PrEP, 324 (32)	Pearson chi-square *p*-value	1878 (22)
**Service delivery model**							
Clinics	692 (96)	27 (4)	**< 0.001**	578 (86)	91 (14)	**< 0.001**	1388 (9)
Youth zones	38 (58)	27 (42)		81 (39)	127 (61)		273 (56)
TVET colleges	35 (71)	14 (29)		26 (28)	67 (72)		142 (57)
High schools	8 (30)	19 (70)		9 (19)	39 (81)		75 (77)
**Age categories**							
15–19 years	79 (79)	21 (21)	**0.01**	170 (61)	110 (39)	**0.002**	380 (34)
20–24 years	298 (88)	40 (12)		524 (71)	214 (29)		1076 (24)
25–29 years	236 (93)	18 (7)		n/a	n/a		254 (7)
30–35 years	160 (95)	8 (5)		n/a	n/a		168 (5)

The p-value of 0.05 was considered statistically significant is given in bold

p-values derived using Person Chi-squared test considered the one stage cluster design

Proportions (%) for the columns reported as n/N

Table 4 shows that PrEP initiation among high risk AGYW accounted for 32% (324/1,018; *p* = 0.002), which was significantly higher compared to high risk ABYM, where initiation was at 10% (87/860; *p* = 0.01). Notably, substantial differences in PrEP initiation rates were observed across the different age groups for the high-risk profile participants, with rates decreasing as age increased as shown in Table 4. This inverse relationship between initiation rates and age was consistent for both males (*p* = 0.01) and females (*p* = 0.002).

### PrEP Adherence Rates Among AGYW and ABYM − 1, 3, 6 Months

**Fig. 6:**
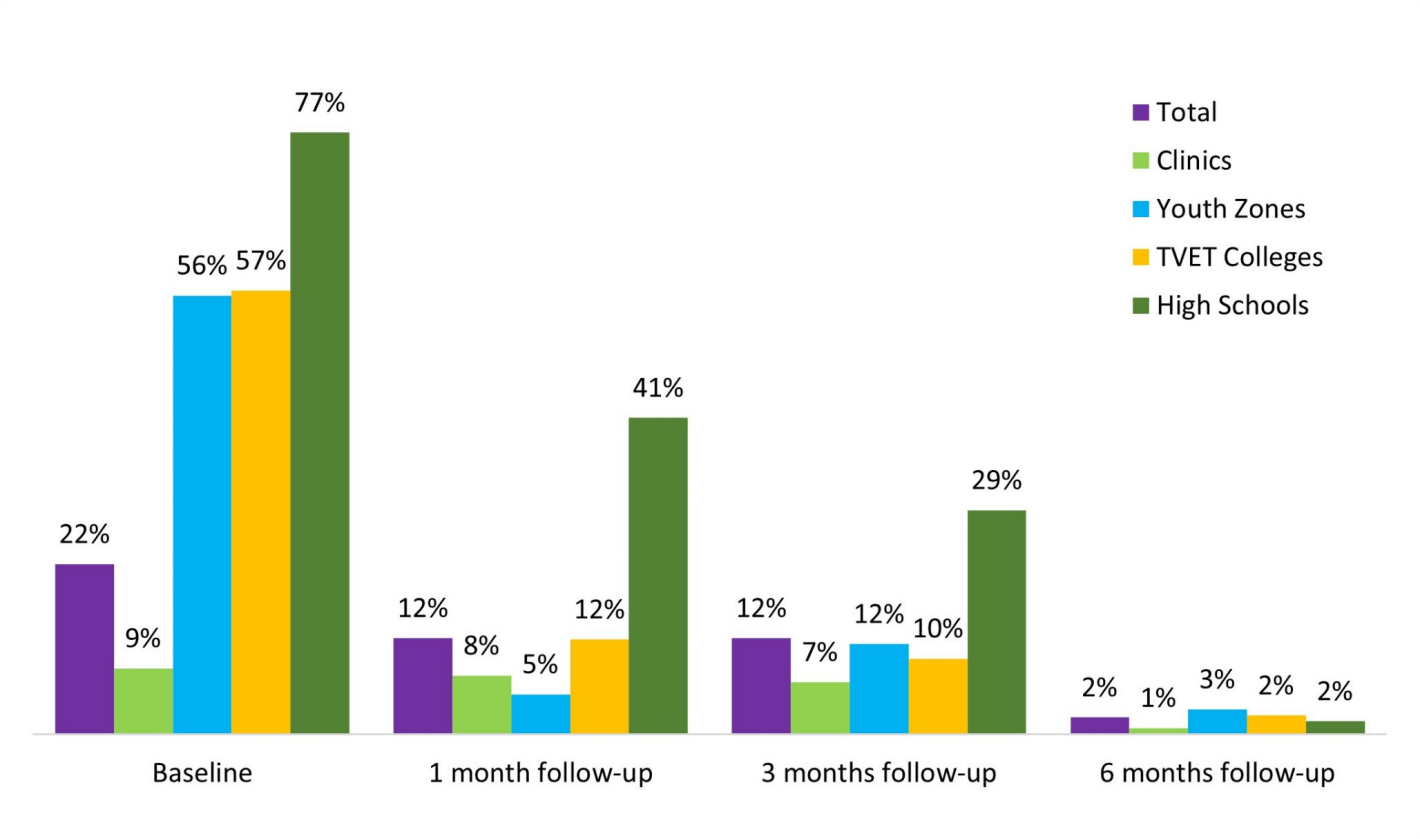
PrEP uptake and adherence rates among the high-risk profile participants (*n* = 412) by SDPs and follow-up intervals, KwaZulu-Natal, South Africa, 2022

Adherence to PrEP after initiation was low in this cohort over the 6-month follow-up period, as indicated in Fig. 6. At baseline, the initial uptake rate was 28% (781/2,772) and 22% (411/1,878) among the high risk for HIV infection. However, there was an over 8-fold reduction in PrEP adherence rate by the 1-month follow-up (12%, 51/412) including the one new PrEP initiator who had not initiated at baseline. The PrEP adherence rate remained consistently low at the 3-month follow-up (12%, 51/412) and declined further at the 6-month follow-up (2%, 9/412) (Chi-square for trend *p* < 0.001).

At the 1-month follow-up, 143 participants (18%) out of the 781 initiates continued PrEP, 19 participants (2%) discontinued, and 5 new participants started PrEP who had not initiated at baseline. Additionally, 1,467 participants (78%) of those with a high-risk profile (1,878) were missed during this period (Fig. 3). PrEP continuation at 3-month (19%, 151/786) was comparable to the 1-month follow-up (19%, 148/786), however, the participants changed status between starting on, discontinuing and restarting PrEP. Continuity on PrEP at 3-month follow-up decreased (7%, 57/786), and 12% (93/786) restarted PrEP after missing or having discontinued at 1-month follow-up. A small proportion (1%, 10/786) discontinued PrEP and about 78% (1464/1878) of the high-risk profile participants were missed during this period. At 6-month follow-up continuity was even lower at 3% (27/786) and 16% (129/786) restarted PrEP after discontinuing or missing at 3-month follow-up.

Table 5 presents the socio-demographic characteristics including service delivery models of the participants for the different follow-up times stratified by PrEP adherence. Participants who initiated PrEP at schools and youth zones are more likely to adhere to PrEP at 3 months (*p* = 0.04) and 6 months (*p* = 0.01).

**Table 5:** Socio-demographic characteristics at different follow-up times by PrEP initiation, KwaZulu Natal, August 2021 to July 2022

	**1-month follow-up**							
Variable	Total (N1 = 190)		On PrEP (N2 = 148)		Off PrEP (N3 = 42)			
Demographic variables	n	% (95% CI)	n	% (95% CI)	n	% (95% CI)	F-value	p-value
**Ethnicity**								
**N**_**1**_ **= 189; N**_**2**_ **= 148; N**_**3**_ **= 41**								
Black African	187	98.4 (93.7–99.8)	148	100	39	95.1 (79.9–99.9)	9.2	**0.02**
Coloured	1	0.5 (0.1–3.2)	0	0	1	2.4 (0.5–10.6)		
Other	1	0.5 (0.1–3.2)	0	0	1	2.4 (0.5–10.6)		
**Sex**								
Male	37	19.5 (9.5–35.8)	30	20.3 (9.6–37.9)	7	16.7 (6.9–35.0)	0.5	0.48
Female	153	80.5 (64.2–90.5)	118	79.7 (62.1–90.4)	35	83.3 (65.0–93.1)		
**Age categories**								
15–19 years	130	68.4 (46.7–84.3)	109	73.7 (57.8–85.1)	21	50.0 (9.2–90.8)	1.3	0.30
20–24 years	57	30.0 (14.9–51.3)	36	24.3 (13.4–40.0)	21	50.0 (9.2–90.8)		
25–29 years	2	1.1 (0.2–5.3)	2	1.4 (0.3–6.8)	0	0		
30–35 years	1	0.5 (0.0–6.0)	1	0.7 (0.1–7.2)	0	0		
**Highest education attained**								
**N**_**1**_ **= 189; N**_**2**_ **= 148; N**_**3**_ **= 41;**								
Primary education	1	0.5 (0.1–4.6)	0	0	1	2.4 (0.3–15.9)	5.3	0.05
High school education	18	99.5 (95.4–99.9)	148	100	40	97.6 (84.1–99.7)		
Tertiary	–	–	–	–	–	–		
Prefer not to answer	–	–	–	–	–	–		
**Marital status**								
Married (living together)	1	0.5 (0.0–6.0)	1	0.7 (0.1–7.2)	0	0	0.8	0.47
Married (living separately)	1	0.5 (0.0–6.0)	1	0.7 (0.1–7.2)	0	0		
Cohabiting	3	1.6 (0.3–8.5)	1	0.7 (0.1–7.2)	2	4.8 (0.4–40.2)		
Dating (living separately)	107	56.3 (49.1–63.3)	82	55.4 (50.1–60.6)	25	59.5 (34.4–80.5)		
Single	77	40.5 (30.4–51.6)	62	41.9 (33.8–50.4)	15	35.7 (15.8–62.2)		
Prefer not to answer	1	0.5 (0.0–6.0)	1	0.7 (0.0–10.6)	0	0		
**Food insecurity**								
Low	164	86.3 (84.8–87.7)	128	86.5 (85.3–87.6)	36	85.7 (77.4–91.3)	0.9	0.41
Medium	16	8.4 (5.9–11.9)	12	8.1 (5.4–12.0)	4	9.5 (3.8–21.8)		
High	2	1.1 (0.3–3.6)	1	0.7 (0.1–6.3)	1	2.4 (0.5–10.7)		
Prefer not to answer	8	4.2 (2.1–8.2)	7	4.7 (2.5–8.7)	1	2.4 (0.5–10.7)		
**Household income**								
R0–999	152	80.0 (73.9–85.0)	118	79.7 (73.8–84.6)	34	81.0 (59.1–92.6)	0.7	0.54
R1000–4999	10	5.3 (3.8–7.3)	6	4.1 (2.4–6.8)	4	9.5 (3.2–25.0)		
R5000+	1	0.5 (0.0–6.0)	1	0.7 (0.1–7.2)	0	0		
Don’t know	20	10.5 (8.4–13.1)	17	11.5 (10.1–13.0)	3	7.1 (1.4–29.0)		
Prefer not to answer	7	3.7 (1.5–8.5)	6	4.1 (1.9–8.3)	1	2.4 (0.2–25.6)		
**Received child grant**								
No	42	22.1 (15.3–30.9)	33	22.3 (14.5–32.8)	9	21.4 (13.4–32.4)	2.9	0.13
Yes	135	71.1 (60.4–79.8)	102	68.9 (55.8–79.6)	33	78.6 (67.6–86.6)		
Don’t know	13	6.8 (4.4–10.4)	13	8.8 (5.8–13.1)	0	0		
**Received disability grant**								
**N**_**1**_ **= 180; N**_**2**_** = 138; N**_**3**_ **= 42;**								
No	161	89.4 (83.2–93.6)	123	89.1 (77.8–95.1)	38	90.5 (77.8–96.3)	0.0	0.84
Yes	19	10.6 (6.4–16.8)	15	10.9 (4.9–22.2)	4	9.5 (3.7–22.2)		
**Received social grant**								
**N**_**1**_ **= 189; N**_**2**_ **= 148; N**_**3**_ **= 41**								
No	89	47.1 (37.6–56.8)	69	46.6 (34.9–58.7)	20	48.8 (37.6–60.1)	0.5	0.69
Yes	84	44.4 (36.0–53.2)	65	43.9 (34.8–53.4)	19	46.3 (32.6–60.7)		
Don’t know	15	7.9 (3.9–15.4)	13	8.8 (4.3–17.2)	2	4.9 (1.2–17.7)		
Prefer not to answer	1	0.5 (0.0–6.8)	1	0.7 (0.1–8.0)	0	0		
**Service Delivery point**								
Clinics	17	8.9 (2.2–29.6)	10	6.8 (1.4–27.6)	7	16.7 (3.2–54.5)	1.9	0.18
Youth Zones	29	15.3 (2.9–52.1)	18	12.2 (3.3–35.7)	11	26.2 (2.3–84.2)		
High Schools	128	67.4 (36.9–87.9)	109	73.7 (48.3–89.3)	19	45.2 (6.8–90.3)		
TVET colleges	16	8.4 (1.8–32.0)	11	7.4 (1.1–37.4)	5	11.9 (3.2–35.3)		

	**3-month follow-up**							
Variable	Total (N4 = 190)		On PrEP (N5 = 148)		Off PrEP (N6 = 42)			
Demographic variables	n	% (95% CI)	n	% (95% CI)	n	% (95% CI)	F-value	p-value
**Ethnicity**								
Black African	189	99.5 (96.4–99.9)	150	100	39	97.5 (78.8–99.8)	3.3	0.10
Coloured	1	0.5 (0.1–3.6)	0	0	1			
Other	–	–	–	–	–			
**Sex**								
Male	34	17.9 (8.8–32.9)	28	18.7 (9.0–34.7)	6	15.0 (7.5–27.7)	1.0	0.35
Female	156	82.1 (67.1–91.2)	122	81.3 (65.3–91.0)	34	85.0 (72.3–92.5)		
**Age categories**								
15–19 years	129	67.9 (55.2–78.4)	103	68.7 (54.7–79.9)	26	65.0 (47.6–79.2)	0.5	0.63
20–24 years	57	30.0 (18.6–44.5)	43	28.7 (16.6–44.8)	14	35.0 (20.8–52.4)		
25–29 years	2	1.1 (0.2–5.3)	2	1.3 (0.3–6.8)	0	0		
30–35 years	2	1.1 (0.2–5.3)	2	1.3 (0.3–6.8)	0	0		
**Highest education attained**								
**N**_**4**_ **= 189; N**_5_ **= 148; N**_**6**_ **= 41**								
Primary education	**–**	–	–	–	–	–		
High school education	185	97.9 (92.9–99.4)	145	97.3 (91.2–99.2)	40	100	0.6	0.52
Tertiary	3	1.6 (0.5–5.1)	3	2.0 (0.6–6.4)	0	0		
Prefer not to answer	1	0.5 (0.1–4.8)	1	0.7 (0.1–5.8)	0	0		
**Marital status**								
Married (living together)	–	–	–	–	–	–		
Married (living separately)	1	0.5 (0.1–5.2)	1	0.7 (0.1–6.6)	0	0	0.3	0.83
Cohabiting	1	0.5 (0.1–5.2)	1	0.7 (0.1–6.6)	0	0		
Dating (living separately)	115	60.5 (54.8–66.0)	89	59.3 (53.2–65.2)	26	65.0 (48.0–78.9)		
Single	70	36.8 (30.2–44.1)	56	37.3 (29.3–46.2)	14	35.0 (21.1–52.0)		
Prefer not to answer	3	1.6 (0.3–7.2)	3	2.0 (0.4–9.4)	0	0		
**Food insecurity**								
Low	168	88.4 (85.3–91.0)	133	88.7 (84.3–91.9)	35	87.5 (64.9–96.4)	0.3	0.76
Medium	15	7.9 (5.3–11.6)	12	8.0 (5.4–11.7)	3	7.5 (1.9–25.8)		
High	2	1.1 (0.2–4.8)	1	0.7 (0.1–5.8)	1	2.5 (0.3–20.3)		
Prefer not to answer	5	2.6 (1.5–4.6)	4	2.7 (1.2–5.9)	1	2.5 (0.3–20.3)		
**Household income**								
R0–999	154	81.1 (74.1–86.5)	120	80.0 (74.1–84.8)	34	85.0 (68.9–93.5)	0.4	0.77
R1000–4999	12	6.3 (3.7–10.5)	9	6.0 (3.9–9.1)	3	7.5 (2.8–18.4)		
R5000+	1	0.5 (0.1–5.2)	1	0.7 (0.1–6.6)	0	0		
Don’t know	15	7.9 (5.1–12.0)	13	8.7 (6.1–12.1)	2	5.0 (1.0–21.7)		
Prefer not to answer	8	4.2 (1.8–9.4)	7	4.7 (1.7–12.0)	1	2.5 (0.2–21.2)		
**Received child grant**								
No	40	21.1 (17.0–25.8)	30	20.0 (15.7–25.1)	10	25.0 (10.9–47.6)	2.3	0.16
Yes	138	72.6 (66.5–78.1)	108	72.0 (66.2–77.2)	30	75.0 (52.4–89.1)		
Don’t know	12	6.3 (4.5–8.7)	12	8.0 (6.2–10.3)	0	0		
**Received disability grant**								
**N**_**4**_ **= 183; N**_**5**_ **= 143; N**_**6**_ **= 40**								
No	155	84.7 (77.0–90.1)	125	87.4 (76.0–93.8)	30	75.0 (55.9–87.7)	2.2	0.17
Yes	28	15.3 (9.9–23.0)	18	12.6 (6.2–24.0)	10	25.0 (12.4–44.1)		
**Received social grant**								
No	94	49.5 (367–62.3)	75	50.0 (36.8–63.3)	19	47.5 (32.2–63.2)	4.3	**0.03**
Yes	85	44.7 (36.4–53.4)	64	42.7 (34.9–50.8)	21			
Don’t know	11	5.8 (2.2–14.3)	11	7.3 (3.1–16.5)	0	52.5 (36.8–67.8)		
Prefer not to answer	–		–		–	0		
**Service Delivery point**								
Clinics	29	15.3 (7.0–30.2)	22	14.7 (5.6–33.1)	7	17.5 (5.6–42.9)	4.4	0.04
Youth Zones	31	16.3 (7.4–32.1)	18	12.0 (5.2–25.2)	13	32.5 (15.8–55.2)		
High Schools	115	60.5 (42.3–76.3)	99	66.0 (45.6–81.8)	16	40.0 (26.4–55.3)		
TVET colleges	15	7.9 (2.0–26.1)	11	7.3 (1.7–26.5)	4	10.0 (3.2–26.9)		

	**6–month follow–up**							
Variable	Total (N7 = 182)		On PrEP (N8 = 155)		Off PrEP (N9 = 27)			
Demographic variables	n	% (95% CI)	n	% (95% CI)	n	% (95% CI)	F-value	p-value
**Ethnicity**								
Black African	180	99.5 (96.4–99.9)	154	99.4 (96.4–99.9)	26	96.3 (81.0–99.9)	0.17	0.86
Coloured	1	0.5 (0.1–3.7)	1	0.6 (0.1–3.6)	0	0		
Other	–	0.5 (0.1–3.7)	–	–	–	3.7 (0.1–19.0)		
**Sex**								
Male	30	16.5 (11.4–22.7)	28	18.1 (12.4–25.0)	2	7.4 (0.9–24.3)	1.9	0.17
Female	152	83.5 (77.3–88.6)	127	81.9 (75.0–87.6)	25	92.6 (75.7–99.1)		
**Age categories**								
15–19 years	106	58.2 (50.7–65.5)	92	59.4 (51.2–67.2)	14	51.9 (31.9–71.3)	1.2	0.72
20–24 years	68	37.4 (30.3–44.8)	56	36.1 (28.6–44.2)	12	44.4 (25.5–64.7)		
25–29 years	5	2.8 (0.1–6.3)	4	2.6 (0.7–6.5)	1	3.7 (0.1–19.0)		
30–35 years	3	1.7 (0.3–4.7)	3	1.9 (0.4–5.6)	0	0		
**Highest education attained**								
Primary education	–	–	–	–	–	–		
High school education	174	95.6 (91.5–98.1)	147	94.8 (90.1–97.7)	27	100	1.3	0.60
Tertiary	7	3.9 (1.6–7.8)	7	4.5 (1.8–9.1)	0	0		
Prefer not to answer	1	0.6 (0.0–3.0)	1	0.7 (0.0–3.5)	0	0		
**Marital status**								
Married (living together)	–	–	–	–	–	–		
Married (living separately)	–	–	–	–	–	–		
Cohabiting	3	1.7 (0.3–4.7)	2	1.3 (0.2–4.6)	1	3.7 (0.1–19.0)	1.5	0.44
Dating (living separately)	119	65.4 (58.0–72.3)	100	64.5 (56.4–72.0)	19	70.4 (49.8–86.2)		
Single	59	32.4 (25.7–39.7)	52	33.6 (26.2–41.6)	7	25.9 (11.1–46.3)		
Prefer not to answer	1	0.5 (0.1–3.7)	1	0.7 (0.0–3.5)	0	0		
**Food insecurity**								
Low	158	86.8 (81.0–91.4)	134	86.5 (80.0–91.4)	24	88.9 (70.8–97.6)	0.9	0.82
Medium	19	10.4 (6.4–15.8)	16	10.3 (6.0–16.2)	3	11.1 (2.4–29.2)		
High	1	0.6 (0.0–3.7)	1	0.6 (0.1–3.6)	0	0		
Prefer not to answer	4	2.2 (0.6–5.5)	4	2.6 (0.7–6.5)	0	0		
**Household income**								
R0–999	150	82.4 (76.1–87.7)	125	80.7 (73.5–86.5)	25	92.6 (75.7–99.1)	2.9	0.73
R1000–4999	8	4.4 (1.9–8.5)	8	5.2 (2.3–9.9)	0	0		
R5000+	1	0.6 (0.0–3.0)	1	0.7 (0.0–3.5)	0	0		
Don’t know	19	10.4 (6.4–15.8)	17	11.0 (6.5–17.0)	2	7.4 (0.9–24.3)		
Prefer not to answer	4	2.2 (0.6–5.5)	4	2.6 (0.7–6.5)	0	0		
**Received child grant**								
No	39	21.4 (15.7–28.1)	31	20.0 (14.0–27.2)	8	29.6 (13.8–50.2)	2.5	0.34
Yes	135	74.2 (67.2–80.4)	116	74.8 (67.2–81.5)	19	70.4 (49.8–86.2)		
Don’t know	8	4.4 (1.9–8.5)	8	5.2 (2.3–9.9)	0	0		
**Received disability grant**								
**N**_**7**_ **= 175; N**_**8**_ **= 148; N**_**9**_ **= 27**								
No	153	87.4 (81.6–91.6)	128	86.5 (79.9–91.5)	25	92.6 (75.7–99.1)	0.8	0.30
Yes	22	12.6 (8.0–18.4)	20	13.5 (8.5–20.1)	2	7.4 (0.9–24.3)		
**Received social grant**								
No	87	47.8 (40.4–55.3)	76	49.0 (40.9–57.2)	11	40.7 (22.4–61.2)	1.4	0.60
Yes	83	45.6 (38.2–53.1)	68	43.9 (35.9–52.1)	15	55.6 (35.3–74.5)		
Don’t know	12	6.6 (3.5–11.2)	11	7.1 (3.6–12.3)	1	3.7 (0.1–19.0)		
Prefer not to answer	–	–	–	–	–	–		
**Service Delivery point**								
Clinics	22	12.1 (7.7–17.7)	16	10.3 (6.0–16.2)	6	22.2 (8.6–42.3)	11.1	0.01
Youth Zones	42	23.1 (17.2–29.9)	31	20.0 (14.0–27.2)	11	40.7 (22.4–61.2)		
High Schools	94	51.7 (44.1–59.1)	87	56.1 (47.9–64.1)	7	25.9 (11.1–46.3)		
TVET colleges	24	13.2 (8.6–19.0)	21	13.6 (8.6–20.0)	3	11.1 (2.4–29.2)		

The p-value of 0.05 was considered statistically significant is given in bold

p-values derived using Pearson Chi-squared test and considered the one stage cluster design

Proportions (%) for the columns reported as n/N (except if data is missing - denominator added in the n column) and the associated 95% CI;* CI* Confidence Interval

## Discussion

This study aims to improve our understanding of effective and feasible oral PrEP delivery models to enhance uptake and adherence among adolescent girls and young women (AGYW) and adolescent boys and young men (ABYM) in KwaZulu-Natal province, South Africa. Our findings show that PrEP initiation rate is higher among females compared to males (38% vs. 13%). Similar trends have been reported in studies by Schaefers et al. (2021) and Shamu et al. (2021) [51, 52]. Conversely, research from Kenya suggests men were more likely to start PrEP than women [53]. The low PrEP initiation and adherence rates among ABYM underscores a significant disparity between potential beneficiaries and actual users. Dovel et al. (2020) attributes the low uptake of HIV services among men and boys to multifaceted factors including harmful gender norms, institutional barriers, and limited entry points to health services; moreover, health policies often prioritize females of reproductive age over males [54]. Hamilton et al. (2023) emphasises the need for youth-focused systemic interventions targeting at-risk adolescents, such as sexual minority males and adults in high-incidence areas, as a lasting solution to ending HIV epidemic [55]. Culturally sensitive and gender-transformative interventions could shift norms and improve ABYM’s PrEP initiation and adherence.

Disparities in PrEP initiation and adherence rates are evident across different Service Delivery Points (SDPs). Clinics exhibit lower PrEP initiation rates compared to high schools, TVET colleges, and community-based youth zones. Previous studies show higher PrEP uptake in routine community-based drop-in SDPs compared to public clinics and private clinics [34]. The authors emphasized that adolescents prefer community-based, youth-friendly PrEP delivery SDPs like schools, due to stigma associated with facility-based services. They highlight the need for targeted interventions and differentiated SDPs as crucial for improving PrEP uptake and adherence.

Youth-friendly clinics, particularly those that minimise room-to-room movement, are preferred for PrEP delivery. Integrated models that incorporate PrEP into family planning, maternal and child health (MCH), and sexual reproductive health (SRH) services provide a convenient, one-stop location for PrEP initiation, especially for AGYW [34, 56]. The DREAMS initiative [33] and community-based approaches, such as mobile health clinic and adolescent-friendly healthcare services [34], help build trust between providers and clients, thereby enhancing PrEP initiation and adherence. Additionally, integrating PrEP with SRH services is highly valued among sexually active adolescents and young adults [57]. Research by Kakande et al. (2023) suggests that ABYM prefer convenient, on-demand, and individualised PrEP delivery options [58]. Strengthening social connectedness through consistent interactions, shared experiences, and supportive networks with providers and peers can further boost PrEP initiation and adherence among ABYM [58]. Therefore, expanding PrEP services to non-traditional settings, such as community-based organizations, high schools, and TVET colleges, holds significant potential for improving PrEP access among young people in South Africa [51].

In the current study, distance to SDPs emerged as a significant factor influencing PrEP uptake. This is consistent with findings by Sullivan et al. (2019), highlighting challenges such as long distances, waiting times, inadequate infrastructure, and shortages of healthcare providers [59]. Strategies are needed to simplify PrEP delivery through differentiated SDP models such as mobile clinics situated near schools. Expanding PrEP delivery through initiatives like drop-in centres, home-based services, and telemedicine-assisted strategies can further facilitate PrEP initiation [60].

Redesigning PrEP delivery through innovative methods is crucial to overcoming barriers faced by adolescents and young adults. Involving non-traditional lay providers, such as young women already on PrEP, can create a more relatable and welcoming environment. Moreso, establishing a friendly supportive atmosphere that offer comprehensive, confidential, and free services, such as multi-month drug refills, peer-delivered PrEP refills, and amenities like free Wi-Fi, has proven effective in promoting PrEP initiation [61]. These strategies can significantly enhance the feasibility and adoption of novel PrEP delivery approaches.

Our findings emphasize the significant role of age in PrEP initiation, with younger adolescents (20–24 years) more likely to start PrEP compared to older age groups. This aligns with observations that ABYM show higher PrEP initiation rates than older men, emphasizing their preference for on-demand PrEP for HIV prevention [58]. Factors contributing to this trend include increased awareness and acceptance of newer HIV prevention strategies among adolescents and young adults.

Findings from our study also highlights that adolescents using contraceptives are less likely to initiate PrEP, highlighting the importance of integrating SRH services with PrEP provision for AGYW. Delivery of PrEP and hormonal contraception via mobile clinics has proven effective in reaching this population [57]. Adolescents with multiple sexual partners are more inclined to initiate PrEP, reflecting their recognition of HIV infection risk and proactive approach to protection.

Engaging men in activities that promote health-seeking behaviour can increase demand for health services and improve sexual risk perception, enhancing PrEP awareness and acceptance. Behavioural interventions such as school-based information campaigns and comprehensive sex education are critical in promoting PrEP uptake among adolescents. Successful PrEP roll-out requires consideration of personal and environmental factors influencing uptake and adherence. Multilevel interventions should address social and structural drivers to inspire PrEP initiation and reduce barriers effectively.

### Strengths and Limitations

This study provides essential insights into optimal service delivery models for initiating and continuing oral PrEP among the target population. It offers valuable data to South African policymakers and implementers on PrEP uptake rates, preferred delivery models, and target demographics, supporting more effective PrEP-based HIV prevention strategies.

However, the study has several potential limitations. Selection bias may have occurred as participants were recruited from various service delivery points (facilities, communities, and schools). Adolescents with severe health conditions, caregiving responsibilities, or from lower socio-economic backgrounds might not have been represented adequately, especially during the COVID- 19 pandemic when some participants may have been absent. COVID- 19-related disruptions to healthcare systems may also have affected data completeness, with clinic records potentially lacking updated information for patients who missed appointments. The study team collaborated with healthcare providers to identify and retrieve missing patient data to mitigate this.

Additionally, challenges in reaching participants for follow-up interviews may have influenced the test-retest reliability assessment, leading to a wide window period for data collection post-baseline (within two weeks). Efforts were made to initiate follow-up interviews promptly after baseline questionnaire completion to minimize potential biases. While this study provides valuable insights, researchers should be mindful of these limitations when interpreting findings and consider strategies to enhance participant inclusivity and data reliability in future research efforts.

## Conclusion

Preliminary findings from this study offer crucial insights for enhancing PrEP uptake and adherence among adolescents and young adults in KwaZulu-Natal, South Africa. Females are more likely to initiate PrEP than males, revealing a significant gap in PrEP access for men. Younger adolescents are also more likely to start PrEP compared to older ones, indicating that interventions should prioritise service delivery in schools and community youth zones. Traditional clinic-based models may need refining to improve PrEP initiation rates.

Expanding PrEP services to non-traditional settings and addressing barriers such as HIV stigma, lack of awareness, potential side effects, and geographical distance can increase PrEP initiation and adherence rates, thereby reducing the HIV burden among adolescents and young adults. These findings offer crucial evidence for policymakers to strengthen PrEP roll-out policies and guidelines for this demography in South Africa. Effective monitoring of PrEP adherence and HIV self-testing is essential. Additionally, implementing strategies to address gender-based violence and provide mental health support can further promote PrEP initiation and adherence, alleviate pressure on health systems, and increase participation among adolescents and young adults.

Identifying barriers to PrEP access and offering convenient, straightforward service delivery approaches that foster social connectedness can be highly beneficial. Training peer leaders or co-facilitators for PrEP education sessions and partnering with community leaders and matriarchs to build trust are effective engagement options.

## Electronic Supplementary Material

Below is the link to the electronic supplementary material.

Supplementary Material 1

## Data Availability

The datasets used and/or analyzed during the current study available from the corresponding author on reasonable request.

## Appendix

See Table 6.

**Table 6:** Risk assessment screening

Questions for Participant (*Consider offering PrEP)				
#	Question	Coding	Coding Value	Score*
601	In the past 6 months: How many people did you have vaginal or anal sex with?	0 1* 2+ (two or more)* Don’t know* Prefer not to answer*	0 1 2 98 99	0 4 7 7 7
602	In the past 6 months: Did you use a condom every time you had sex?	Yes No* Don’t know* Prefer not to answer*	1 2 98 99	0 10 10 10
603	Engaged in sex in exchange of money or other favors?	Yes* No Don’t know* Prefer not to answer*	1 2 98 99	5 0 5 5
604	In the past 6 months: Did you have a sexually transmitted infection?	Yes* No Don’t know* Prefer not to answer*	1 2 98 99	7 0 7 7
605	Shared needles while engaging in intravenous drug use?	Yes* No Don’t know Prefer not to answer*	1 2 98 99	3 0 0 3
606	Do you have a sexual partner who has HIV?	Yes* No Don’t know* Prefer not to answer*	1 2 98 99	8 0 8 8
607	If ‘Yes’: Has he or she been on therapy for 6 or more months?	Yes No* Don’t know* Prefer not to answer	1 2 98 99	0 3 3 0
608	If ‘Yes: has the therapy suppressed viral load?	Yes No* Don’t know* Prefer not to answer	1 2 98 99	0 3 3 0
609	PrEP stands for pre-exposure prophylaxis. It is a single daily pill that protects you from getting infected with HIV. It works when you take it before you are exposed to HIV. Are you willing to consider PrEP?	Yes No Don’t know Prefer not to answer	1 2 98 99	

*Scoring adapted from Smith et al., [50] (42)

## References

[1] UNAIDS. People living with HIV: AIDSinfo; 2024. [30 March 2024]. Available from: http://aidsinfo.unaids.org/

[2] UNAIDS, Country factsheets. South Africa 2023-HIV and AIDS Estimates [30 March 2024]. Available from: https://www.unaids.org/en/regionscountries/countries/southafrica

[3] Gomez A, Malone S, Prasad R, Gangaramany A, Croucamp Y, Mulhausen J, et al. Understanding HIV prevention in high-risk adolescent girls and young women in two South African provinces. South Afr Health Rev. 2019;15:167–72.

[4] Pulerwitz J, Mathur S, Woznica D. How empowered are girls/young women in their sexual relationships? relationship power, HIV risk, and partner violence in Kenya. PLoS One. 2018;13(7):e0199733. 30024908 10.1371/journal.pone.0199733 PMC6053148

[5] de Oliveira T, Kharsany AB, Graf T, Cawood C, Khanyile D, Grobler A, et al. Transmission networks and risk of HIV infection in KwaZulu-Natal, South Africa: a community-wide phylogenetic study. Lancet HIV. 2017;4(1):e41-50. 27914874 10.1016/S2352-3018(16)30186-2 PMC5479933

[6] Van Damme W, Kober K, Kegels G. Scaling-up antiretroviral treatment in Southern African countries with human resource shortage: how will health systems adapt? Soc Sci Med. 2008;66(10):2108–21. 18329774 10.1016/j.socscimed.2008.01.043

[7] Dunbar MS, Kripke K, Haberer J, Castor D, Dalal S, Mukoma W, et al. Understanding and measuring uptake and coverage of oral pre-exposure prophylaxis delivery among adolescent girls and young women in sub-Saharan Africa. Sex Health. 2018;15(6):513–21. 30408431 10.1071/SH18061 PMC6429961

[8] South African National Department of Health. Guidelines for expanding combination prevention and treatment options for sex workers: Oral pre-exposure prophylaxis (PrEP) and test and treat (T&T). Final Draft. Pretoria, South Africa.2016.

[9] Braksmajer A, Senn TE, McMahon J. The potential of pre-exposure prophylaxis for women in violent relationships. AIDS Patient Care STDs. 2016;30(6):274–81. 27286296 10.1089/apc.2016.0098 PMC4913495

[10] O’Malley TL, Hawk ME, Egan JE, Krier SE, Burke JG. Intimate partner violence and pre-exposure prophylaxis (PrEP): a rapid review of current evidence for women’s HIV prevention. AIDS Behav; 2019. 10.1007/s10461-019-02743-x 31776819

[11] Cabral A, Ngure JMB, Velloza K, Odoyo J. Intimate partner violence and self-reported pre-exposure prophylaxis interruptions among HIV-negative partners in HIV serodiscordant couples in Kenya and Uganda. J Acquir Immune Defic Syndr. 2018;77(2):154–9. 29076883 10.1097/QAI.0000000000001574 PMC5762272

[12] Palanee-Phillips T, Roberts ST, Reddy K, Govender V, Naidoo L, Siva S, et al. Impact of partner-related social harms on women’s adherence to the Dapivirine vaginal ring during a phase III trial. J Acquir Immune Defic Syndr. 2018;79(5):580–9. 30239426 10.1097/QAI.0000000000001866 PMC6231955

[13] Roberts ST, Haberer J, Celum C, Mugo N, Ware NC, Cohen CR, et al. Intimate partner violence and adherence to HIV pre-exposure prophylaxis (PrEP) in African women in HIV serodiscordant relationships: a prospective cohort study. J Acquir Immune Defic Syndr. 2016;73(3):313–22. 27243900 10.1097/QAI.0000000000001093 PMC5065369

[14] Celum CL, Delany-Moretlwe S, Baeten JM, van der Straten A, Hosek S, Bukusi EA, et al. HIV pre-exposure prophylaxis for adolescent girls and young women in Africa: from efficacy trials to delivery. J Int AIDS Soc. 2019;22(Suppl 4):e25298. 31328444 10.1002/jia2.25298 PMC6643076

[15] Ajayi AI, Mudefi E, Yusuf MS, Adeniyi OV, Rala N, Goon DT. Low awareness and use of pre-exposure prophylaxis among adolescents and young adults in high HIV and sexual violence prevalence settings. Medicine. 2019;98(43):e17716. 31651904 10.1097/MD.0000000000017716 PMC6824740

[16] South African National Department of Health. PrEP implementation pack: South Africa 2016–2017. South Africa: Pretoria; 2017.

[17] Emmanuel G, Folayan M, Undelikwe G, Ochonye B, Jayeoba T, Yusuf A, et al. Community perspectives on barriers and challenges to HIV pre-exposure prophylaxis access by men who have sex with men and female sex workers access in Nigeria. BMC Public Health. 2020;20(1):69. 31941469 10.1186/s12889-020-8195-x PMC6964078

[18] Pilgrim N, Jani N, Mathur S, Kahabuka C, Saria V, Makyao N, et al. Provider perspectives on PrEP for adolescent girls and young women in Tanzania: the role of provider biases and quality of care. PLoS One. 2018;13(4):e0196280. 29702659 10.1371/journal.pone.0196280 PMC5922529

[19] Geibel S, Hossain SM, Pulerwitz J, Sultana N, Hossain T, Roy S, et al. Stigma reduction training improves healthcare provider attitudes toward, and experiences of, young marginalized people in Bangladesh. J Adolesc Health: Off Publ Soc Adolesc Med. 2017;60(2s2):S35-44. 10.1016/j.jadohealth.2016.09.026 28109339

[20] Sam-Agudu NA, Folayan MO, Ezeanolue EE. Seeking wider access to HIV testing for adolescents in sub-Saharan Africa. Pediatr Res. 2016;79(6):838–45. 26882367 10.1038/pr.2016.28

[21] World Health Organization. Global standards for quality health-care services for adolescents: a guide to implement a standards-driven approach to improve the quality of health care services for adolescents. Switzerland: Geneva; 2015.

[22] Corneli A, Wang M, Agot K, Ahmed K, Lombaard J, Van Damme L. Perception of HIV risk and adherence to a daily, investigational pill for HIV prevention in FEM-PrEP. J Acquir Immune Defic Syndr. 2014;67(5):555–63. 25393942 10.1097/QAI.0000000000000362

[23] Macintyre K, Rutenberg N, Brown L, Karim A. Understanding perceptions of HIV risk among adolescents in KwaZulu-Natal. AIDS Behav. 2004;8(3):237–50. 15475673 10.1023/B:AIBE.0000044072.71361.b3

[24] O’Byrne P, Orser L, Haines M. Active-Offer Nurse-Led PrEP (PrEP-RN). Referrals: analysis of uptake rates and reasons for declining. AIDS Behav; 2019. 10.1007/s10461-019-02745-9 PMC7156352 31773443

[25] Murnane PM, Celum C, Mugo N, Campbell JD, Donnell D, Bukusi E, et al. Efficacy of preexposure prophylaxis for HIV-1 prevention among high-risk heterosexuals: subgroup analyses from a randomized trial. AIDS. 2013;27(13):2155–60. 24384592 10.1097/QAD.0b013e3283629037 PMC3882910

[26] Marrazzo JM, Ramjee G, Richardson BA, Gomez K, Mgodi N, Nair G, et al. Tenofovir-based preexposure prophylaxis for HIV infection among African women. N Engl J Med. 2015;372(6):509–18. 25651245 10.1056/NEJMoa1402269 PMC4341965

[27] Van Damme L, Corneli A, Ahmed K, Agot K, Lombaard J, Kapiga S, et al. Preexposure prophylaxis for HIV infection among African women. N Engl J Med. 2012;367(5):411–22. 22784040 10.1056/NEJMoa1202614 PMC3687217

[28] Adeyeye AO, Stirratt MJ, Burns DN. Engaging men in HIV treatment and prevention. Lancet. 2018;392(10162):2334–5. 30527600 10.1016/S0140-6736(18)32994-5

[29] Staveteig S, Croft TN, Kampa KT, Head SK. Reaching the ‘first 90’: gaps in coverage of HIV testing among people living with HIV in 16 African countries. PLoS One. 2017;12(10):e0186316. 29023510 10.1371/journal.pone.0186316 PMC5638499

[30] UNAIDS. Blind spot: reaching out to men and boys. Addressing a blind spot in the response to HIV, Geneva. Switzerland: UNAIDS; 2017.31. Afri-Can Forum 2: Johannesburg, South Africa. 16–18 February 2015. BMC infectious diseases. 2016;16 Suppl 2:315. 10.1186/s12879-016-1466-6 PMC4943497 27410689

[31] Mukumbang FC. Leaving no man behind: how differentiated service delivery models increase men’s engagement in HIV care. Int J Health Policy Manag. 2020;15(2):109–21. 10.34172/ijhpm.2020.32 PMC7947905 32610748

[32] Orr N, Hajiyiannis H, Myers L, Makhubele MB, Matekane T, Delate R, et al. Development of a national campaign addressing South African men’s fears about HIV counseling and testing and antiretroviral treatment. J Acquir Immune Defic Syndr. 2017;74(Suppl 1):S69-73. 27930614 10.1097/QAI.0000000000001204 PMC5147035

[33] George G, Cawood C, Puren A, Khanyile D, Gerritsen A, Govender K, et al. Evaluating DREAMS HIV prevention interventions targeting adolescent girls and young women in high HIV prevalence districts in South Africa: protocol for a cross-sectional study. BMC Womens Health. 2020;20(1):7. 31948429 10.1186/s12905-019-0875-2 PMC6966796

[34] Ramraj T, Chirinda W, Jonas K, Govindasamy D, Jama N, McClinton Appollis T, Zani B, Mukumbang FC, Basera W, Hlongwa M, Turawa EB, Mathews C, Nicol E. Service delivery models that promote linkages to PrEP for adolescent girls and young women and men in sub-Saharan Africa: a scoping review. BMJ Open. 2023;13(3):e061503. 10.1136/bmjopen-2022-061503. PMID: 36972966; PMCID: PMC10069497. 36972966 10.1136/bmjopen-2022-061503 PMC10069497

[35] Fox MP, Pascoe S, Huber AN, et al. Adherence clubs and decentralized medication delivery to support patient retention and sustained viral suppression in care: results from a cluster randomized evaluation of differentiated ART delivery models in South Africa. PLOS Med. 2019;16:e1002874. 31335865 10.1371/journal.pmed.1002874 PMC6650049

[36] Subedar H, Barnett S, Chaka T, Dladla S, Hagerman E, Jenkins S, et al. Tackling HIV by empowering adolescent girls and young women: a multisectoral, government led campaign in South Africa. BMJ. 2018;363:k4585. 10.1136/bmj.k4585. 30530572 10.1136/bmj.k4585 PMC6284473

[37] The Aurum Institute. Men’s Health Month: Aurum partners to implement campaigns supporting men living with HIV. [Available from: https://www.auruminstitute.org/component/content/article/28-blog/aurum-news/455-men-s-health-month-mina-and-coach-mpi lo-f ight - the- s t igma- for-men- l iving-with-hiv?Itemid = 101. Date accessed: 26 Nov. 2021.].

[38] Fleming PJ, Colvin C, Peacock D, et al. What role can gender-transformative programming for men play in increasing men’s HIV testing and engagement in HIV care and treatment in South Africa? Cult Health Sex. 2016;18:1251–64. 27267890 10.1080/13691058.2016.1183045 PMC5030173

[39] Njuguna N, Akolo C, Anzala O, Baeten JM, Heffron R, Mugo NR, Bateganya M. Differentiated service delivery models for maintaining HIV treatment and prevention services during crisis and disease outbreaks: lessons from the COVID-19 pandemic. Curr HIV/AIDS Rep. 2024;21(5):257–63. 10.1007/s11904-024-00703-2. 39052142 10.1007/s11904-024-00703-2

[40] UNAIDS. Global HIV & AIDS statistics—2020 fact sheet. UNAIDS. Retrieved from UNAIDS website; 2020.

[41] WHO. (2020). Preventing HIV through safe voluntary medical male circumcision for adolescent boys and men in generalized HIV epidemics: recommendations and key considerations. Retrieved from https://www.who.int/publications/i/item/978-92-4-000854-0 32986340

[42] Mayer CM, Owaraganise A, Kabami J, Kwarisiima D, Koss CA, Charlebois ED, Kamya MR, Petersen ML, Havlir DV, Jewell BL, et al. Distance to clinic is a barrier to PrEP uptake and visit attendance in a community in rural Uganda. J Int AIDS Soc. 2019;22(4):e25276. 10.1002/jia2.25276. PMID: 31037845; PMCID: PMC6488759. 31037845 10.1002/jia2.25276 PMC6488759

[43] Gill K, Johnson L, Dietrich J, Myer L, Marcus R, Wallace M, Bekker LG. Acceptability, feasibility, and preferences for oral and long-acting injectable HIV pre-exposure prophylaxis in adolescent girls and young women in South Africa. Clin Infect Dis. 2021;73(5):737–42.

[44] Eakle R, Bourne A, Mbogua J, Mutanha N, Rees H. Exploring acceptability of oral PrEP prior to implementation among female sex workers in South Africa. J Int AIDS Soc. 2018;21(2):e25081. 29457868 10.1002/jia2.25081 PMC5817972

[45] Nicol E, Ramraj T, Hlongwa M, Basera W, Jama N, et al. Strengthening health system’s capacity for pre-exposure prophylaxis for adolescent girls and young women and adolescent boys and young men in South Africa (SHeS’Cap–PrEP) protocol for a mixed methods study in KwaZulu-Natal South Africa. PLoS One. 2022;17(3):e0264808. 10.1371/journal.pone.0264808. 35298487 10.1371/journal.pone.0264808 PMC8929690

[46] Nunn AS, Brinkley-Rubinstein L, Oldenburg CE, Mayer KH, Mimiaga M, Patel R, et al. Defining the HIV pre-exposure prophylaxis care continuum. AIDS. 2017;31(5):731–4. 28060019 10.1097/QAD.0000000000001385 PMC5333727

[47] Beesham I, Joseph Davey DL, Beksinska M, Bosman S, Smit J, Mansoor LE. Daily oral Pre-exposure prophylaxis (PrEP) continuation among women from Durban, South Africa, who initiated PrEP as standard of care for HIV prevention in a clinical trial. AIDS Behav. 2022;26(8):2623–31. Epub 2022 Feb 5. PMID: 35122575; PMCID: PMC9252967. 35122575 10.1007/s10461-022-03592-x PMC9252967

[48] Harris PA, Taylor R, Thielke R, et al. Research electronic data capture (REDCap)-a metadata-driven methodology and workflow process for providing translational research informatics support. J Biomed Inf. 2009;42:377–81. 10.1016/j.jbi.2008.08.010 PMC2700030 18929686

[49] Myburgh H, Murphy JP, van Huyssteen M, et al. Implementation of an electronic monitoring and evaluation system for the antiretroviral treatment programme in the cape winelands district, South Africa: a qualitative evaluation. PLoS One. 2015;10:e0127223. 25966294 10.1371/journal.pone.0127223 PMC4429075

[50] Smith DK, et al. Development of a clinical screening index predictive of incident HIV infection among men who have sex with men in the united States. JAIDS J Acquir Immune Defic Syndr. 2012;60(4):421–7. 22487585 10.1097/QAI.0b013e318256b2f6

[51] Schaefer R, Schmidt HM, Ravasi G, Mozalevskis A, Rewari BB, Lule F, Yeboue K, Brink A, Konath NM, Sharma M, Seguy N. Adoption of guidelines on and use of oral pre-exposure prophylaxis: a global summary and forecasting study. Lancet HIV. 2021;8(8):e502–10. 34265283 10.1016/S2352-3018(21)00127-2 PMC8332196

[52] Shamu S, Shamu P, Khupakonke S, Farirai T, Chidarikire T, Guloba G, Nkhwashu N. Pre-exposure prophylaxis (PrEP) awareness, attitudes and uptake willingness among young people: gender differences and associated factors in two South African districts. Global Health Act. 2021;14(1):1886455. 10.1080/16549716.2021.1886455 PMC7899653 33606603

[53] Olilo WA, Petersen ML, Koss CA, Wafula E, Kwarisiima D, Kadede K, Clark TD, Cohen CR, Bukusi EA, Kamya MR, Charlebois ED. Pre-exposure prophylaxis (PrEP) uptake among older individuals in rural Western Kenya. JAIDS J Acquir Immune Defic Syndr. 2019;82(4):e50-3. 31490343 10.1097/QAI.0000000000002150 PMC6831040

[54] Dovel K, Dworkin SL, Cornell M, et al. Gendered health institutions: examining the organization of health services and men’s use of HIV testing in Malawi. J Int AIDS Soc. 2020;23(S2):e25517. 32589346 10.1002/jia2.25517 PMC7319160

[55] Hamilton DT, Wang LY, Hoover KW, Smith DK, Delaney KP, Li J, Hoyte T, Jenness SM, Goodreau SM. Potential contribution of PrEP uptake by adolescents 15–17 years old to achieving the ending the HIV epidemic incidence reduction goals in the US South. PLoS One. 2023;18(11):e0288588. 10.1371/journal.pone.0288588. PMID: 37943869; PMCID: PMC10635552. 37943869 10.1371/journal.pone.0288588 PMC10635552

[56] Celum C, Mgodi N, Bekker L-G, Hosek S, Donnell D, Anderon P et al. Adherence 3 months after PrEP initiation among young African women in HPTN 082. Conference on retroviruses and opportunistic infections (CROI); March 4–7; Seattle, WA; 2019.

[57] Rousseau E, Bekker LG, Julies RF, Celum C, Morton J, Johnson R, Baeten JM, O’Malley G. A community-based mobile clinic model delivering PrEP for HIV prevention to adolescent girls and young women in cape town, South Africa. BMC Health Serv Res. 2021;21(1):888. 10.1186/s12913-021-06920-4. PMID: 34454505; PMCID: PMC8403426. 34454505 10.1186/s12913-021-06920-4 PMC8403426

[58] Kakande A, Ssemata AS, Muhumuza R, et al. Preferences for oral PrEP dosing among adolescent boys and young men in three sub-Saharan African countries. PLoS One. 2023;18(10):e0285132. 10.1371/journal.pone.0285132. PMID: 37812644; PMCID: PMC10561834. 37812644 10.1371/journal.pone.0285132 PMC10561834

[59] Sullivan PS, Mena L, Elopre L, et al. Implementation strategies to increase PrEP uptake in the South. Curr HIV/AIDS Rep. 2019;16:259–69. 10.1007/s11904-019-00447-4. 31177363 10.1007/s11904-019-00447-4 PMC7117066

[60] O’Malley G, Barnabee G, Mugwanya K. Scaling-up PrEP delivery in sub-Saharan Africa: what can we learn from the scale-up of art? Curr HIV/AIDS Rep. 2019;16:141–50. 10.1007/s11904-019-00437-6. 30796608 10.1007/s11904-019-00437-6 PMC6469867

[61] Haberer JE, Mugo N, Baeten JM, Pyra M, Bukusi E, Bekker L-G. PrEP as a lifestyle and investment for adolescent girls and young women in sub-Saharan Africa. J Int Assoc Provid AIDS Care (JIAPAC). 2019. 10.1177/2325958219831011. 30776954 10.1177/2325958219831011 PMC6748528

