# Supplementary material for: Assessing PrEP Initiation and Adherence Among High-Risk, Sexually Active Adolescents and Young Adults: A Population-Based Prospective Study Across Diverse Service Delivery Models in a High HIV Prevalent District in South Africa
Source: AIDS Behav. 2025 May 2;29(8):2588–609. doi: 10.1007/s10461-025-04719-6 (PMC12378780; doi:10.1007/s10461-025-04719-6)
Supplement: Supplementary file 1 — Supplementary Material 1 [file 10461_2025_4719_MOESM1_ESM.docx]

Supplement 1: Socio-demographic characteristics at baseline by PrEP uptake in uMgungundlovu district between August 2021 to July 2022

| **Variable** | | **Total (N_1_=2,772)** | | | | **Initiated PrEP (N_2_=781)** | | | | **Did not initiate PrEP (N_3_=1991)** | | |  |
| --- | --- | --- | --- | --- | --- | --- | --- | --- | --- | --- | --- | --- | --- |
| **Demographic variables** | | **n** | | **% (95% CI)** | | **n** | | **% (95% CI)** | | **n** | **% (95% CI)** | **p-value** |  |
| **Nationality**  South African  Other SADC  Other African  Outside Africa | | 2751  18  2  1 | | 99.2 (98.3-99.7)  0.6 (0.3-1.4)  0.1 (0.0-0.2)  0.0 (0.0-0.3) | | 778  2  0  1 | | 99.6 (98.5-99.9)  0.3 (0.0-1.7)  0  0.1 (0.0-1.1) | | 1973  16  2  0 | 99.1 (97.9-99.6)  0.8 (0.4-1.8)  0.1 (0.0-0.3)  0 | 0.16 |  |
| **Ethnicity**  **N_1_=2,768; N_2_=779; N_3_=1,989**  Black African  Coloured  White  Indian/Asian  Other | | 2748  14  2  2  1 | | 99.3 (98.0-99.7)  0.5 (0.2-1.3)  0.1 (0.0-0.5)  0.1 (0.0-0.2)  0.0 (0.0-0.3) | | 777  1  0  0  1 | | 99.7 (98.3-100)  0.1 (0.0-0.9)  0  0  0.1 (0.0-0.9) | | 1971  13  2  2  0 | 99.1 (97.5-99.7)  0.7 (0.3-1.7)  0.1 (0.0-0.6)  0.1 (0.0-0.3)  0 | 0.12 |  |
| Age (years) | | 2772 | | 21 (19-24) | | 781 | | 19 (17-22) | | 1991 | 22 (19-24) | ***<0.001 **** |  |
| **Sex**  Male  Female | | 1078  1694 | | 38.9 (30.1-48.5)  61.1 (51.5-69.9) | | 141  640 | | 18.0 (12.9-24.7)  82.0 (75.4-87.1) | | 937  z1054 | 47.1 (37.4-56.9)  52.9 (43.1-62.6) | ***<0.001*** |  |
| **Age categories**  15-19 years  20-24 years  25-29 years  30-35 years | | 857  1384  337  194 | | 30.9 (19.4-45.4)  49.9 (40.3-59.6)  12.2 (8.8-16.5)  7.0 (4.2-11.4) | | 402  339  31  9 | | 51.5 (27.4-74.9)  43.4 (22.6-66.8)  4.0 (2.3-6.9)  1.2 (0.5-2.9) | | 455  1045  306  185 | 22.9 (16.0-31.5)  52.5 (47.8-57.1)  15.4 (11.9-19.7)  9.3 (5.9-14.3) | ***<0.01*** |  |
| **Highest education attained**  ***N_1_=2,769; N_2_=779; N_3_=1,990***  No education  Primary education  High school education  Tertiary | | 2  33  2533  196 | | 0.1 (0.0-0.3)  1.2 (0.9-1.6)  91.5 (84.0-95.6)  7.1 (3.2-15.0) | | 0  9  748  21 | | -  1.2 (0.7-2.0)  96.0 (92.1-98.0)  2.7 (1.1-6.4) | | 2  24  1785  175 | 0.1 (0.0-0.4)  1.2 (0.9-1.7)  89.7 (81.0-94.7)  8.8 (4.0-18.1) | ***0.02*** |  |
| **Currently studying**  ***N_1_=2,766; N_2_=779; N_3_=1,987***  No  Yes | | 1241  1523 | | 44.9 (36.7-53.3)  55.1 (46.7-63.2) | | 198  580 | | 25.4 (15.5-38.9)  74.5 (61.0-84.4) | | 1043  943 | 52.5 (43.9-60.9)  47.5 (39.1-56.0) | ***<0.001*** |  |
| **Marital status**  ***N_1_=2,763; N_2_=778; N_3_=1,985***  Married (living together)  Married (living separately)  Cohabiting  Dating (living separately)  Single  Prefer not to answer | | 19  9  103  1947  66  19 | | 0.7 (0.3-1.5)  0.3 (0.1-0.8)  3.7 (2.5-5.6)  70.5 (65.7-74.9)  24.1 (19.5-29.5)  0.7 (0.4-1.1) | | 1  3  16  519  232  7 | | 0.1 (0.0-1.1)  0.4 (0.1-1.2)  2.1 (0.9-4.7)  66.7 (55.8-76.1)  29.8 (19.6-42.5)  0.9 (0.4-2.0) | | 18  6  87  1428  434  12 | 0.9 (0.4-2.0)  0.3 (0.1-1.0)  4.4 (2.8-6.7)  71.9 (68.1-75.5)  21.9 (18.3-25.9)  0.6 (0.3-1.1) | 0.10 |  |
| **Transport to facility**  ***N_1_=2,759; N_2_=779; N_3_=1,980***  On foot  Public transport (taxi/bus)  Private transport (own/other)  Other (Res/TBHIV care) | | 1579  856  304  10 | | 57.2 (41.671.6)  31.0 (19.1-46.2)  11.0 (6.9-17.1)  0.4 (0.1-1.3) | | 480  155  135  5 | | 61.6 (44.7-76.1)  19.9 (13.1-29.0)  17.3 (9.0-30.9)  0.6 (0.3-1.5 | | 1099  701  169  5 | 55.5 (37.6-72.1)  35.4 (21.1-52.9)  8.5 (6.0-12.0)  0.3 (0.0-1.5) | ***0.03*** |  |
| **Time to get to the facility**  ***N_1_=2,768; N_2_=781; N_3_=1,987***  Less than 30 minutes  30-60 minutes  More than an hour  Don’t know | | 2018  670  59  15 | | 72.9 (65.2-79.4)  24.2 (18.1-31.5)  2.1 (1.4-3.3)  0.5 (0.3-1.1) | | 626  133  13  7 | | 80.2 (73.4-85.6)  17.0 (11.2-25.0)  1.7 (0.9-3.0)  0.9 (0.5-1.6) | | 1392  537  46  8 | 70.1 (60.6-78.1)  27.0 (19.9-35.6)  2.3 (1.3-4.0)  0.4 (0.1-1.2) | ***0.01*** |  |
| **Service Delivery Point**  Clinics  Youth Zones  Schools | | 1833  472  467 | | 66.1 (47.2-81.0)  17.0 (10.0-27.6)  16.9 (7.5-33.7) | | 165  275  341 | | 21.1 (9.1-41.8)  35.2 (20.7-53.1)  43.7 (24.2-65.4) | | 1668  197  126 | 83.8 (67.9-92.7)  9.9 (4.3-21.1)  6.3 (1.9-19.2) | ***<0.001*** |  |
| **Socio-economic variables** | | | | | | | | | |  | |  |  |
| **Worked past 12 months**  ***N_1_=2,754; N_2_=775; N_3_=1,979***  Never worked  Once in a while  Most months  Every month  Don’t know | | 1607  262  263  585  25 | | 58.4 (51.4-65.0)  9.5 (5.5-16.1)  9.6 (7.5-12.1)  21.2 (15.5-28.4)  0.9 (0.7-1.2) | | 539  21  28  174  9 | | 69.6 (64.0-74.6)  2.7 (1.7-4.2)  3.6 (1.9-6.8)  22.5 (18.6-26.9)  1.2 (0.7-1.9) | | 1068  241  235  411  16 | 54.0 (45.9-61.8)  12.2 (7.3-19.7)  11.9 (9.7-14.5)  20.8 (13.8-30.1)  0.8 (0.5-1.4) | ***<0.001*** |  |
| **Household income**  ***N_1_=2,765; N_2_=780; N_3_=1,985***  R0-999  R1000-4999  R5000+  Don’t know  Prefer not to answer | | 1947  418  118  179  103 | | 70.4 (61.2-78.2)  15.1 (9.9-22.4)  4.3 (2.1-8.5)  6.5 (5.0-8.4)  3.7 (2.7-5.1) | | 628  58  6  56  32 | | 80.5 (76.3-84.1)  7.4 (5.0-10.9)  0.8 (0.2-3.6)  7.2 (5.2-9.8)  4.1 (3.0-5.6) | | 1319  360  112  123  71 | 66.5 (56.0-75.5)  18.1 (12.1-26.3)  5.6 (2.9-10.7)  6.2 (4.5-8.4)  3.6 (2.5-5.1) | ***<0.01*** |  |
| **Received child grant**  ***N_1_=2,767; N_2_=780; N_3_=1,987***  No  Yes  Don’t know | | 923  1752  85 | | 33.4 (26.1-41.5)  63.3 (55.1-70.8)  3.1 (2.1-4.5) | | 184  570  26 | | 23.6 (19.7-28.0)  73.1 (69.7-76.2)  3.3 (1.8-6.1) | | 739  1182  59 | 37.2 (29.1-46.1)  59.5 (50.5-67.9)  3.0 (1.9-4.6) | ***<0.01*** |  |
| **Sexual experience and other related factors** | | | | | | | | | | | | | |
| **Age at sexual debut**  ***N_1_=2,206; N_2_=493; N_3_=1713***  ≤14years  15-17years  ≥18years  Don’t know | | 149  1009  987  42 | | 6.8 (4.9-9.2)  45.7 (40.3-51.2)  44.7 (39.4-50.2)  1.9 (1.0-3.8) | | 29  236  226  1 | | 5.9 (2.8-12.12)  47.9 (38.6-57.3)  45.8 (33.2-57.3)  0.2 (0.0-1.7) | | 120  773  761  41 | | 7.0 (5.1-9.6)  45.1 (39.3-51.1)  44.4 (39.4-49.6)  2.4 (1.3-4.4) | 0.26 |
| **First sex experience**  ***N_1_=2206; N_2_=494; N_3_=1712***  Wanted  Persuaded  Raped  Don’t know | | 1766  388  39  3 | | 80.1 (75.4-84.0)  17.6 (13.6-22.5)  1.8 (1.2-2.5)  0.1 (0.0-0.5) | | 384  93  13  1 | | 77.7 (73.6-81.4)  18.8 (15.4-22.8)  2.6 (1.4-4.9)  0.2 (0.0-2.0) | | 1382  295  26  2 | | 80.7 (75.4-85.2)  17.2 (12.6-23.0)  1.5 (1.0-2.4)  0.1 (0.0-0.5) | 0.44 |
| **Sexual partners last year**  ***N_1_=2011; N_2_=436; N_3_=1575***  One partner  Two-three partners  Four or more partners  Don’t know | | 1125  638  226  13 | | 55.9 (47.564.1)  31.7 (25.9-38.1)  11.2 (9.6-13.2)  0.6 (0.2-2.0) | | 309  102  25  0 | | 70.9 (64.7-76.4)  23.4 (18.6-29.1)  5.7 (3.7-8.9)  0 | | 816  536  201  13 | | 51.8 (43.0-60.6)  34.0 (27.8-40.9)  12.8 (10.9-14.8)  0.8 (0.3-2.4) | ***<0.001*** |
| **Sex with main partner**  ***N_1_=2182; N_2_=481; N_3_=1701***  No  Yes | | 242  1905 | | 11.1 (6.9-17.3)  87.3 (81.6-91.4) | | 36  439 | | 7.5 (5.7-9.7)  91.3 (88.8-93.2) | | 206  1466 | | 12.1 (7.4-19.2)  86.2 (79.7-90.9) | 0.06 |
| **Sex with additional partner**  ***N_1_=2179; N_2_=480; N_3_=1699***  No  Yes | | 1434  729 | | 65.8 (60.3-71.0)  33.5 (28.2-39.1) | | 363  116 | | 75.6 (68.2-81.8)  24.2 (18.0-31.7) | | 1071  613 | | 63.0 (57.2-68.6)  36.1 (30.4-42.2) | ***<0.01*** |
| **Use condom in last sexual experience** | | | | | | | | | | | | | 0.86 |
| ***N_1_=2201; N_2_=493; N_3_=1708***  No/Part of the time  Yes  Don’t know | | 1511  676  5 | | 68.7 (65.8-71.4)  30.7 (28.0-33.6)  0.2 (0.1-0.7) | | 337  152  2 | | 68.4 (61.2-74.8)  30.8 (24.8-37.6)  0.4 (0.1-1.8) | | 1174  524  3 | | 68.7 (65.6-71.7)  30.7 (27.6-34.0)  0.2 (0.0-1.0) |  |
| **On contraceptives**  ***N_1_=1690; N_2_=632; N_3_=1058***  No  Yes  Prefer not to answer | | 974  709  7 | | 57.6 (50.9-64.1)  42.0 (35.7-48.5)  0.4 (0.2-0.9) | | 414  217  1 | | 65.5 (55.5-74.3)  34.3 (25.7-44.2)  0.2 (0.0-1.2) | | 560  492  6 | | 52.9 (45.6-60.1)  46.5 (39.6-53.6)  0.6 (0.2-1.5) | ***0.03*** |
